# Inhibition of Heat Shock proteins *HSP90* and *HSP70* induce oxidative stress, suppressing cotton fiber development

**DOI:** 10.1038/s41598-018-21866-0

**Published:** 2018-02-26

**Authors:** Anshulika Sable, Krishan M. Rai, Amit Choudhary, Vikash K. Yadav, Sudhir K. Agarwal, Samir V. Sawant

**Affiliations:** 1 Plant Molecular Biology Laboratory, National Botanical Research Institute, Rana Pratap Marg, Lucknow, 226001 India; 2 Department of Biochemistry, University of Lucknow, Lucknow, 226007 India; 3 Present Address: Fiber and Biopolymer Research Institute (FBRI), Department of Plant and Soil Science, Texas Tech University, Lubbock, Texas 79409 USA; 4 Present Address: Department of Plant Biology, Uppsala Biocenter, Swedish University of Agricultural Sciences, Uppsala, 75007 Sweden

## Abstract

Cotton fiber is a specialized unicellular structure useful for the study of cellular differentiation and development. Heat shock proteins (HSPs) have been shown to be involved in various developmental processes. Microarray data analysis of five *Gossypium hirsutum* genotypes revealed high transcript levels of *GhHSP90* and *GhHSP70* genes at different stages of fiber development, indicating their importance in the process. Further, we identified 26 and 55 members of *HSP90* and *HSP70* gene families in *G. hirsutum*. The treatment of specific inhibitors novobiocin (Nov; HSP90) and pifithrin/2-phenylethynesulfonamide (Pif; HSP70) in *in-vitro* cultured ovules resulted in a fewer number of fiber initials and retardation in fiber elongation. The molecular chaperone assay using bacterially expressed recombinant *GhHSP90-7* and *GhHSP70-8* proteins further confirmed the specificity of inhibitors. HSP inhibition disturbs the H_2_O_2_ balance that leads to the generation of oxidative stress, which consequently results in autophagy in the epidermal layer of the cotton ovule. Transmission electron microscopy (TEM) of inhibitor-treated ovule also corroborates autophagosome formation along with disrupted mitochondrial cristae. The perturbations in transcript profile of HSP inhibited ovules show differential regulation of different stress and fiber development-related genes and pathways. Altogether, our results indicate that HSP90 and HSP70 families play a crucial role in cotton fiber differentiation and development by maintaining cellular homeostasis.

## Introduction

Cotton fiber, one of the longest unicellular cells, provides an ideal platform for studying cellular differentiation, development and cell wall synthesis in the plant system. Cotton fiber development includes four distinct but overlapping stages viz. initiation, elongation, secondary cell wall (SCW) biosynthesis and maturation. Fiber development involves an intricate pattern of transcriptional and translational regulation to facilitate the transition between different stages. An array of genes including transcriptional factors have been reported to play an essential role in fiber initiation and elongation^1,2^. Apart from this, several metabolic processes such as hormonal pathways^2^, sugar metabolism

^3^, secondary metabolites

^4^, H_2_O_2_ balance, etc. are also reported to play a critical role in fiber development. The H_2_O_2_ treatment has been shown to induce cotton fiber initials in *XinFLM* cotton fiber developmental mutant^5^. Furthermore, treatment of cotton ovules with appropriate concentration of H_2_O_2_ prompts fiber elongation via ethylene signaling pathway^6,7^. However, an increase in the H_2_O_2_ concentration beyond optimal level causes oxidative stress, which eventually leads to a decline in the fiber growth^8^. The imbalance in the levels of H_2_O_2_ may cause an adverse effect on the physiology of the organism, by creating stress conditions that may eventually lead to apoptosis^9,10^. The cell has evolved anti-apoptotic molecules such as HSPs that are induced during the heat stress and delay apoptosis in different cell lines^11^.

HSPs are a non-identical group of multi-family proteins that are chaperones in nature, primarily predicted to help in the survival of organisms on exposure to stress^12^. HSPs tend to be an evolutionarily conserved group of proteins throughout the prokaryotes and eukaryotes. These proteins are distributed among five major families viz. HSP100, HSP90, HSP70, HSP60/40, and HSP20 by their molecular weight^13^. All of these families help in maintaining cellular homeostasis and play distinct non-redundant roles in different developmental processes. Out of these families, *HSP90* has been reported to be actively expressed in root and shoot apices^14^, during embryogenesis and pollen development^15^ suggesting their role in different developmental processes. *HSP90* also contributes to buffering phenotypic variations and developmental stability in *Arabidopsis*^16^. HSP90 and HSP70 have been reported to interact with heat shock factors directly and regulate their activity in tomato^17^. HSP70 also helps in organelle-specific protein sorting^18^ and directs proteins to ubiquitin-mediated proteasomal degradation pathways^19^. Down-regulation of *HSP70* in *Arabidopsis* subjects to thermal sensitivity^20^ and developmental defects^21,22^. Additionally, *HSP70*/*HSP90* machinery also plays a role in stomatal closure and response to Abscisic acid in *Arabidopsis*^23^.

HSPs thus seems to play an essential role in different developmental processes and stress conditions in plants^24,25^. Studies on several HSP gene families were carried out in various plant species. *HSP90* family comprises of 7, 9 and 10 members^26,27^ whereas, *HSP70* family consists of 18, 26 and 20 members in *Arabidopsis thaliana*, *Oryza sativa* and *Populus trichocarpa*, respectively^28^. Availability of *G. hirsutum* genome sequence provides the opportunity to explore the HSP gene families in this economically important plant species. Cotton is a field grown plant susceptible to all kind of stresses, there are several reports which point towards the preferential expression of HSPs in different stress conditions, but limited literature is available, indicating towards their involvement in any of its developmental processes^29^. The commercial availability of HSP specific inhibitors provides a novel platform to explore the importance of HSP proteins in cotton fiber development. Few inhibitors are known for HSP90 and HSP70 but none for HSP100 and HSP20. Recently, butyl 3-[2-(2,4-dichlorophenoxy) acetamido] benzoate has been shown to inhibit HSP60/40^30^. In plants, HSP90 inhibitor (Geldanamycin) has been shown to decline the root growth in *Arabidopsis*, suggesting its crucial role in root elongation and thus its suitability for functional studies as HSP90 inhibitor^31^. Further, HSP90 inhibitor, Nov has been reported to interact with the C-terminal domain of HSP90 that constitutes the dimerization interface and co-chaperone binding domain^32^. Similarly, HSP70 inhibitor, Pif^33^ also hinders the co-chaperone and the substrate binding property of HSP70. However, the effect of Pif and Nov in plant system is not analyzed before.

In the present study, we have explored the *HSP90* and *HSP70* gene families in *G. hirsutum* and their role in cotton fiber development. We assessed the effect of inhibition of HSP90 and HSP70 in *in-vitro* ovule culture to study its impact on fiber development. Our results suggest a significant role of HSP70 and HSP90 in maintaining homeostasis during fiber initiation and elongation.

## Results

### Cotton fiber development concurs with high-level expression of HSPs

All the living organisms are equipped with several classes of structurally unrelated molecular chaperones to ensure proper protein folding during stress condition or rapid development. Analysis of previously published microarray gene expression data^34^ on six fiber developmental conditions in five genotypes of *G. hirsutum*, revealed that based on the transcript levels, HSPs are clustered into three distinct clusters (Supplementary Fig. S1). The cluster-II belongs to genes that expressed at high level in almost all the tested developmental stages. The cluster-II consists of different type of HSPs including *HSP90* and *HSP70* genes, indicating their involvement throughout the fiber development. The transcript level of *GhHSP90-7* and *GhHSP70-8* belonging to cluster-II was further validated by qRT-PCR, which showed the high transcript level of both these genes especially during initiation and early elongation (Supplementary Fig. S2).

### Gene family structure of *HSP90* and *HSP70* in *G. hirsutum*

The transcript levels of *GhHSP90* and *GhHSP70* pointed out their possible role in fiber development. Thus we explored gene family structure of these two chaperons. The *HSP90* family in *G. hirsutum* comprises of total 26 members, 13 each from A and D sub-genomes and contain HSP90 and HATPase_c domain (Table 1). The *GhHSP90* family members are distributed on chromosome number (Ch.) 1, 3, 6, 7, 8, 12 and 13 in A sub-genome and on Ch. 1, 2, 3, 6, 7, 8, 12 and 13 in D sub-genome (Table 1). All the homoeologs of *GhHSP90* family members are present concurrently on chromosomes of A and D sub-genomes, except for *GhHSP90-3A* and *GhHSP90-3D* which are present on Ch. A3 and D2 respectively. Further, *GhHSP90-5A.1* and *GhHSP90-5A.2*, *GhHSP90-5D.1* and *GhHSP90-5D.2*, *GhHSP90-7A.1* and *GhHSP90-7A.2*, *GhHSP90-7D.1* and *GhHSP90-7D.2* seem to evolve due to independent duplication event. Thus, the presence of four copies each of *GhHSP90-5* and *GhHSP90-7* in *G. hirsutum* genome indicates their possible evolutionary importance in growth and development of cotton.

**Table 1:** Details of *GhHSP90* gene family.

	**Gene name**	**Gene id**	**Chromosomal position**	**Protein size (aa)**	**Molecular Wt. (KDa)**	**Sub-cellular localization**
A-subgenome	GhHSP90-1A	Gh_A01G0741	ChrA01: 14221053-14223941	699	80.036	Cytoplasmic
	GhHSP90-2A	Gh_A01G0894	ChrA01: 21244688-21249882	835	95.704	ER
	GhHSP90-4A	Gh_A03G0164	ChrA03: 60064701-60070485	698	80.155	Cytoplasmic
	GhHSP90-3A	Gh_A03G0935	ChrA03: 2491019-2494057	832	94.864	Cytoplasmic
	GhHSP90-5A.1	Gh_A06G0030	ChrA06: 223387-227511	804	91.812	ER
	GhHSP90-5A.2	Gh_A06G0031	ChrA06: 229779-233909	805	92.020	ER
	GhHSP90-6A	Gh_A07G1723	ChrA07: 70380260-70385380	797	90.469	Mitochondrial
	GhHSP90-7A.1	Gh_A08G0219	ChrA08: 2276489-2279126	699	80.068	Cytoplasmic
	GhHSP90-7A.2	Gh_A08G0220	ChrA08: 2308805-2311733	699	80.070	Cytoplasmic
	GhHSP90-8A	Gh_A08G0998	ChrA08: 69729838-69731569	318	36.430	Cytoplasmic
	GhHSP90-9A	Gh_A12G2300	ChrA12: 85650149-85653034	703	80.643	Cytoplasmic
	GhHSP90-10A	Gh_A13G0766	ChrA13: 32062236-32067412	752	84.989	Chloroplastic
	GhHSP90-11A	Gh_A13G1098	ChrA13: 61336732-61339412	699	80.143	Cytoplasmic
D-subgenome	GhHSP90-1D	Gh_D01G0761	ChrD01: 10914595-10917558	699	80.022	Cytoplasmic
	GhHSP90-2D	Gh_D01G0932	ChrD01: 15653549-15657679	809	92.490	ER
	GhHSP90-3D	Gh_D02G1319	ChrD02: 43578662-43584441	790	90.159	Cytoplasmic
	GhHSP90-4D	Gh_D03G1421	ChrD03: 42981450-42984339	707	81.116	Cytoplasmic
	GhHSP90-5D.2	Gh_D06G2283	Scaffold4085_D06: 71331-75444	804	92.044	ER
	GhHSP90-5D.1	Gh_D06G2284	Scaffold4085_D06: 64964-69077	804	92.000	ER
	GhHSP90-6D	Gh_D07G1926	ChrD07: 47773815-47778936	797	90.404	Mitochondrial
	GhHSP90-7D.1	Gh_D08G0299	ChrD08: 2913021-2915658	699	80.022	Cytoplasmic
	GhHSP90-7D.2	Gh_D08G0300	ChrD08: 2928411-2931340	699	80.048	Cytoplasmic
	GhHSP90-8D	Gh_D08G1269	ChrD08: 41600905-41603592	704	81.051	Cytoplasmic
	GhHSP90-9D	Gh_D12G2436	ChrD12: 57396182-57399137	704	80.858	Cytoplasmic
	GhHSP90-10D	Gh_D13G0899	ChrD13: 18219499-18224694	723	82.197	Chloroplastic
	GhHSP90-11D	Gh_D13G1363	ChrD13: 42677111-42679747	699	80.139	Cytoplasmic

ER: Endoplasmic Reticulum.

*GhHSP70* family is relatively large consisting of 55 members and characterized by the presence of HSP70 domain. In *Arabidopsis*, the *HSP70* family grouped into HSP70 sub-class and HSP110/SSE sub-class based on their molecular weight^28^. Similarly in *G. hirsutum*, out of 55 members, 43 belong to HSP70 sub-class (19 from A and 24 from D sub-genomes) and 12 (6 each from A and D sub-genomes) belong to HSP110/SSE sub-class. The *GhHSP70* family members are present on all the chromosomes except Ch. 4 and 7 (Table 2). Like *GhHSP90* gene family, all the *GhHSP70* genes are located on respective homologous chromosomes in both the sub-genomes, except for *GhHSP70-3A* and *GhHSP70-3D*, which is located on A2 and D3 respectively. We failed to detect A sub-genome specific homoeologs of five members namely *GhHSP70-5D*, *GhHSP70-6D*, *GhHSP70-11D*, *GhHSP70-16D*, and *GhHSP70-27D* whereas *GhHSP70-26D* is a partial sequence. The homoeolog of *GhHSP70-31A* (present on Ch. A10) was identified on scaffold Gh_Sca004937G04 and named as *GhHSP70-31D* (Table 2).

**Table 2:** Details of *GhHSP70* gene family.

		**Gene name**	**Gene id**	**Chromosomal position**	**Protein size (aa)**	**Molecular Weight (Da)**	**Sub-cellular localization**
HSP70	A-subgenome	GhHSP70-1A	Gh_A01G1923	ChrA01: 99150074-99153161	648	71.216	Cytoplasmic
		GhHSP70-2A	Gh_A02G0073	ChrA02: 594987-598554	704	75.430	Chloroplastic
		GhHSP70-3A	Gh_A02G0951	ChrA02: 39564783−39568727	666	73.381	ER
		GhHSP70-4A	Gh_A03G0353	ChrA03: 6375626-6378707	648	70.824	Cytoplasmic
		GhHSP70-7A	Gh_A05G0823	ChrA05: 8267506-8270108	650	71.113	Cytoplasmic
		GhHSP70-8A	Gh_A06G1477	ChrA06: 98009202-98011384	648	70.997	Cytoplasmic
		GhHSP70-13A	Gh_A09G1469	ChrA09: 67930785-67933441	646	70.869	Cytoplasmic
		GhHSP70-14A	Gh_A09G2326	Scaffold2287_A09: 7896-11002	718	77.299	Chloroplastic
		GhHSP70-15A	Gh_A09G2245	Scaffold2279_A09: 7165-9702	592	64.531	Cytoplasmic/ PM
		GhHSP70-19A	Gh_A10G1292	ChrA10: 67260889-67264010	706	75.703	Chloroplastic
		GhHSP70-21A	Gh_A11G0171	ChrA11: 1610300-1613891	667	73.533	ER
		GhHSP70-22A	Gh_A11G0174	ChrA11: 1644547-1647992	667	73.532	ER
		GhHSP70-23A	Gh_A11G1883	ChrA11: 48505796-48509036	678	72.971	Mitochondrial
		GhHSP70-24A	Gh_A11G2910	ChrA11: 93003683-93006152	647	70.780	Cytoplasmic
		GhHSP70-25A	Gh_A12G0151	ChrA12: 2232912-2235970	677	72.404	Mitochondrial
		GhHSP70-26A	Gh_A12G0152	ChrA12: 2269312-2272374	681	72.814	Mitochondrial
		GhHSP70-29A	Gh_A13G0895	ChrA13: 46842882-46846653	622	69.043	ER
		GhHSP70-30A	Gh_A13G2046	ChrA13: 79817836-79819794	652	71.384	Cytoplasmic
		GhHSP70-31A	Gh_A10G1940	ChrA10: 96749888-96752352	652	71.222	Cytoplasmic
	D-subgenome	GhHSP70-1D	Gh_D01G2180	ChrD01: 60746226-60749258	648	71.204	Cytoplasmic
		GhHSP70-2D	Gh_D02G0088	ChrD02: 631172-634756	704	75.441	Chloroplastic
		GhHSP70-3D	Gh_D03G0811	ChrD03: 27753247-27756364	666	73.383	ER
		GhHSP70-4D	Gh_D03G1221	ChrD03: 39491745-39494905	648	70.924	Cytoplasmic
		GhHSP70-5D	Gh_D03G1225	ChrD03: 39573593-39578627	584	65.563	Cytoplasmic
		GhHSP70-6D	Gh_D03G1549	ChrD03: 44705138-44707084	648	70.806	Cytoplasmic
		GhHSP70-7D	Gh_D05G0943	ChrD05: 7907708-7910317	650	71.174	Cytoplasmic
		GhHSP70-8D	Gh_D06G1814	ChrD06: 58203414-58205599	648	71.011	Cytoplasmic
		GhHSP70-11D	Gh_D08G1192	ChrD08: 38372483-38375636	666	73.252	ER
		GhHSP70-13D	Gh_D09G1479	ChrD09: 42364418-42367075	646	70.871	Cytoplasmic
		GhHSP70-14D	Gh_D09G2036	ChrD09: 47728996-47732064	735	79.022	Chloroplastic
		GhHSP70-15D	Gh_D09G2082	ChrD09: 48164612-48167151	592	64.721	Cytoplasmic/ PM
		GhHSP70-16D	Gh_D09G2084	ChrD09: 48173223-48174926	567	62.652	Cytoplasmic/ PM
		GhHSP70-19D	Gh_D10G1189	ChrD10: 20558563-20561664	706	75.708	Chloroplastic
		GhHSP70-21D	Gh_D11G0181	ChrD11: 1618339-1621899	667	73.532	ER
		GhHSP70-22D	Gh_D11G0184	ChrD11: 1641418-1644817	667	73.451	ER
		GhHSP70-23D	Gh_D11G2087	ChrD11: 29998341-30002431	682	73.337	Mitochondrial
		GhHSP70-24D	Gh_D11G3296	ChrD11: 65856743-65859212	647	70.983	Cytoplasmic
		GhHSP70-25D	Gh_D12G0164	ChrD12: 2116044-2119093	677	72.406	Mitochondrial
		GhHSP70-26D	Gh_D12G0165	ChrD12: 2130556-2132570	354	37.57	Mitochondrial
		GhHSP70-27D	Gh_D12G1067	ChrD12: 36783328-36784989	553	60.936	Cytoplasmic
		GhHSP70-29D	Gh_D13G1136	ChrD13: 33658463-33661024	657	72.809	ER
		GhHSP70-30D	Gh_D13G2447	ChrD13: 60389215-60391173	652	71.384	Cytoplasmic
		GhHSP70-31D	Gh_Sca004937G04	Scaffold4937: 28658-31068	652	71.307	Cytoplasmic
HSP110/SSE	A-subgenome	GhHSP70-9A	Gh_A06G1513	ChrA06: 98810230-98817100	911	101.51	Nuclear/ ER
		GhHSP70-10A	Gh_A08G2507	Scaffold2268_A08: 181657-184884	757	84.780	Nuclear
		GhHSP70-12A	Gh_A09G0792	ChrA09: 54719230-54722953	856	94.427	Cytoplasmic
		GhHSP70-17A	Gh_A10G0130	ChrA10: 1084260-1088698	774	86.965	Nuclear
		GhHSP70-18A	Gh_A10G0712	ChrA10: 12129630-12133303	855	94.337	Cytoplasmic
		GhHSP70-20A	Gh_A10G1717	ChrA10: 91002111-91010963	879	98.502	ER/ Nuclear
	D-subgenome	GhHSP70-9D	Gh_D06G2388	Scaffold4164_D06: 47843-55469	913	101.573	Nuclear/ ER
		GhHSP70-10D	Gh_D08G0244	ChrD08: 2325136-2328250	757	84.872	Nuclear
		GhHSP70-12D	Gh_D09G0795	ChrD09: 32510122-32513840	856	94.599	Cytoplasmic
		GhHSP70-17D	Gh_D10G0137	ChrD10: 1086860-1091313	774	86.814	Nuclear
		GhHSP70-18D	Gh_D10G0674	ChrD10: 7564910-7568532	855	94.195	Cytoplasmic
		GhHSP70-20D	Gh_D10G1993	ChrD10: 55383231-55392087	879	98.686	ER/ Nuclear

ER: Endoplasmic Reticulum.

**Figure 1:**
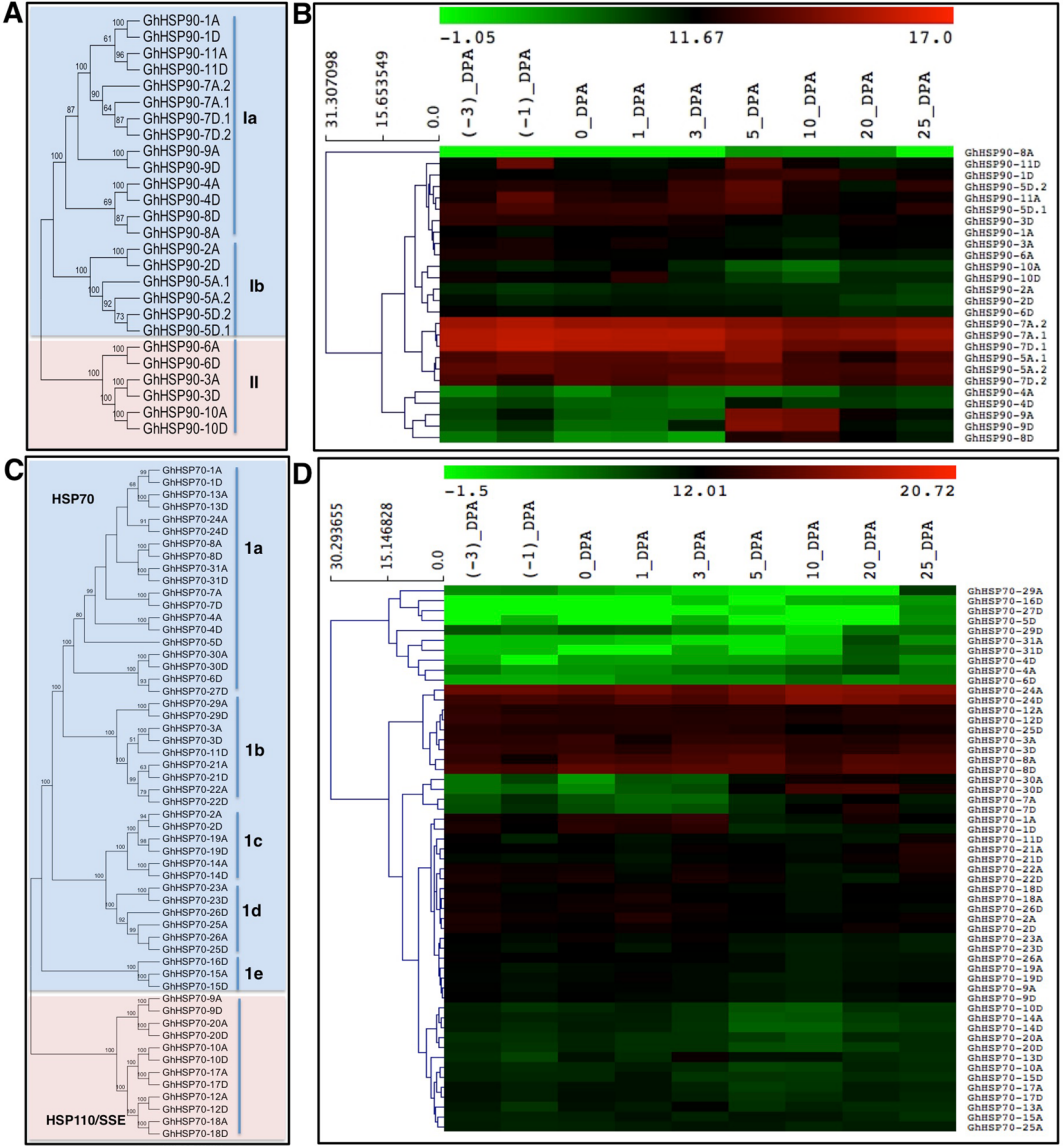
Phylogenetic and transcript level analysis of *HSP90* and *HSP70* members in *G. hirsutum*. (**A**) Phylogenetic tree of *GhHSP90* members. (**B**) Heat map showing transcript levels of *GhHSP90* members at different stages of fiber development. (**C**) Phylogenetic tree of *GhHSP70* members. (**D**) Heat map showing transcript levels of *GhHSP70* members at different stages of fiber development.

Sub-cellular localization prediction indicated that members of both the families were localized either in the cytoplasm or other sub-cellular organelles. Of all the members of GhHSP90 and GhHSP70 sub-class, 16 and 23 members were cytoplasmic. Other members were found to localize either in the ER (six and nine members respectively), chloroplast (two and five members respectively) or in the mitochondria (two and six members respectively) (Tables 1 and 2). Similarly, the members of HSP110/SSE sub-class were also predicted to localize in the cytoplasm (four members), nucleus (four members), ER/nucleus (two members) or the nucleus/ER (two members) (Table 2). The phylogenetic tree showed GhHSP90 family members grouped mainly into two groups with members of each branch having similar sub-cellular localization. The members of the group I were further sub-categorized into Ia (members localized in the cytoplasm) and Ib (members localized in the ER), whereas group II members were predicted to localize in the chloroplast or mitochondria (Fig. 1A). In the case of GhHSP70, the phylogenetic tree bifurcated the two sub-classes on different branches, which further formed groups based on their sub-cellular localization. The HSP70 subclass grouped into Ia, Ib, Ic, Id, and Ie that were predicted to localize into the cytoplasm, ER, chloroplast, mitochondria, and cyto/PM, respectively (Fig. 1C). Whereas, the HSP110/SSE members formed altogether a different cluster II (Fig. 1C).

To probe further into the possible role of *GhHSP90* and *GhHSP70* genes during fiber development, we investigated their transcript levels. Among *GhHSP90* members, *GhHSP90-7* and *GhHSP90-5A* showed higher transcript levels in all the stages of fiber development, suggesting their importance throughout the fiber development. *GhHSP90-2* and *GhHSP90-6D* showed medium transcript levels in all the stages whereas, *GhHSP90-4*, *GhHSP90-9*, and *GhHSP90-8D* showed significant transcript levels in the later stages of development. *GhHSP90-10* was found to express significantly in early stages, and *GhHSP90-8A* has poor transcript levels in all the stages of development (Fig. 1B). Similarly, the members of *GhHSP70* were clustered into genes with low transcript levels in all the stages (*GhHSP70-29*, *GhHSP70-16D*, *GhHSP70-27D*, *GhHSP70-5D*, *GhHSP70-31*, *GhHSP70-4* and *GhHSP70-6D*), genes with high transcript levels in all the stages (*GhHSP70-24*, *GhHSP70-12*, *GhHSP70-25D*, *GhHSP70-3*, and *GhHSP70-8*) and genes with intermediate transcript levels in all the stages (*GhHSP70-10*, *GhHSP70-14*, *GhHSP70-20*, *GhHSP70-13*, *GhHSP70-15*, *GhHSP70-17*, *GhHSP70-25A*, *GhHSP70-23*, *GhHSP70-26A*, *GhHSP70-19* and *GhHSP70-9*) (Fig. 1D). While *GhHSP70-30* and *GhHSP70-7* showed significant transcript level in the later stages, *GhHSP70-1*, *GhHSP70-22*, *GhHSP70-18*, *GhHSP70-26D*, *GhHSP70-2*, *GhHSP70-11D* and *GhHSP70-21* showed higher transcript levels in early stages of fiber development, suggesting their importance in their corresponding stages (Fig. 1D).

### HSP90 and HSP70 activities are essential for the appropriate development of cotton fiber

**Figure 2:**
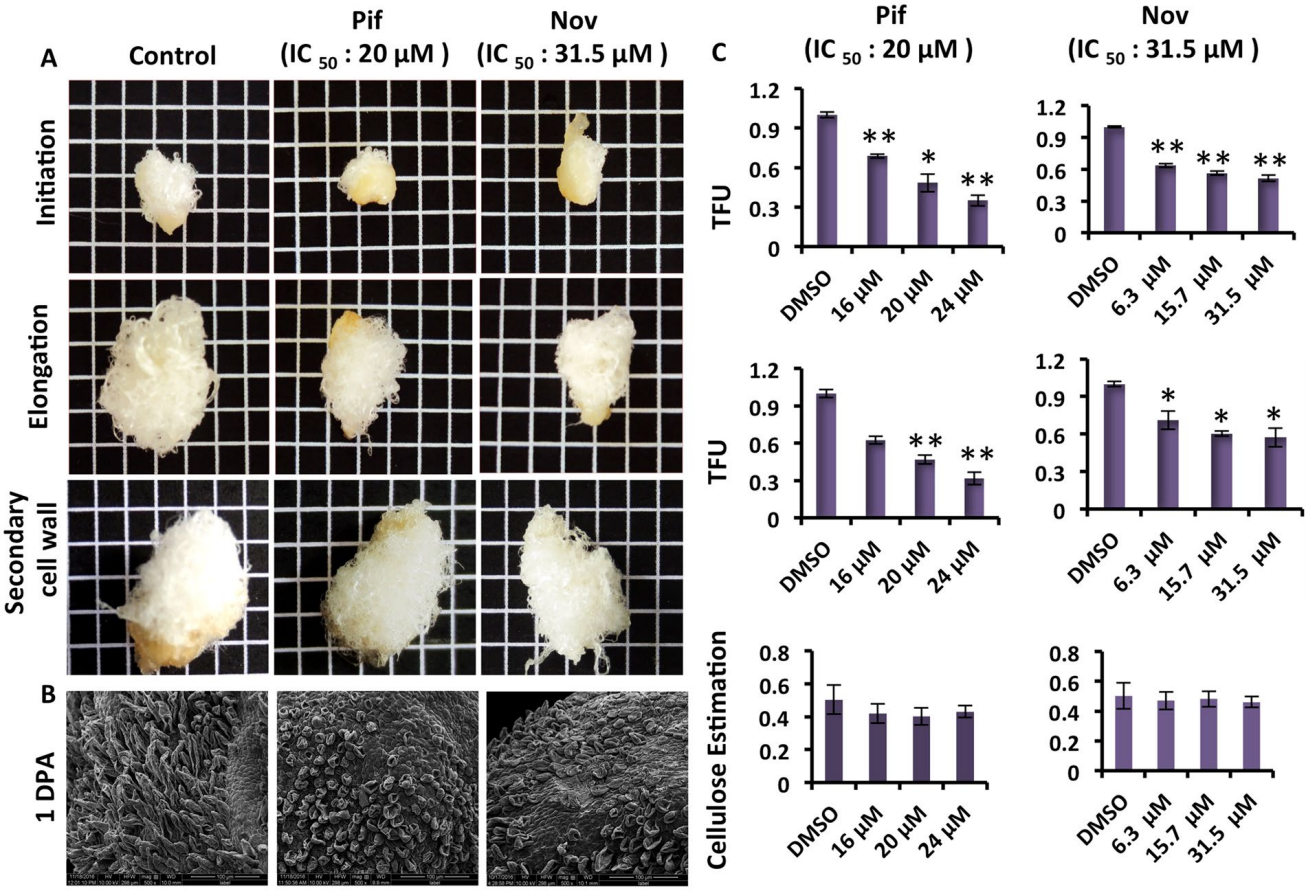
Effect of different HSP inhibitors on *in-vitro* ovule culture at different stages of cotton fiber development. (**A**) Photographs of *in-vitro* cultured ovules in initiation, elongation and SCW stage showing the effect of Pif (HSP70) and Nov (HSP90) inhibitors. (**B**) SEM of control and treated ovules at 1 DPA shows clear decline in fiber growth. (**C**) Quantitative estimation of fiber growth in initiation, elongation and SCW stage at different concentration of Pif and Nov inhibitors (X-axis and Y-axis designates O.D. and concentration of inhibitors, respectively). The asterisks represent statistical significance between three biological and three technical replicates (*p-value ≤ 0.05, **p-value ≤ 0.01).

Nov and Pif are reported inhibitors for HSP90^32^ and HSP70^33^ classes of proteins, respectively. We assessed the role of HSP90 and HSP70 in cotton fiber development by treating developing cotton ovules with Nov and Pif respectively in *in-vitro* ovule culture (Fig. 2A). The varying concentrations of Nov and Pif were used to determine the IC_50_ value (Supplementary Fig. S3). Both the inhibitors showed significant inhibition of fiber development with increasing concentrations as also indicated by decreasing total fiber unit (TFU) (Fig. 2C). This result was further confirmed by the decline in fiber growth observed in scanning electron microscopy (SEM) of inhibitor-treated ovules (Fig. 2B). The inhibition of the fiber development was more pronounced when inhibitors were added either at initiation or at the elongation stage for both Nov and Pif (Fig. 2A). The IC_50_ for Nov for both the initiation and elongation stages was 31.5 µM, while that of Pif for both stages was 20 µM (*p-value ≤ 0.05, **p-value ≤ 0.01). We did not observe any significant inhibition by either Nov or Pif when treated at SCW biosynthesis stage; this is also indicated by no change in cellulose content (Fig. 2A and C). Thus, our results showed that appropriate chaperonic activities of HSP90 and HSP70 are crucial for optimal fiber development at initiation and elongation stages.

### Nov and Pif are inhibitors of cotton HSP90 and HSP70

**Figure 3:**
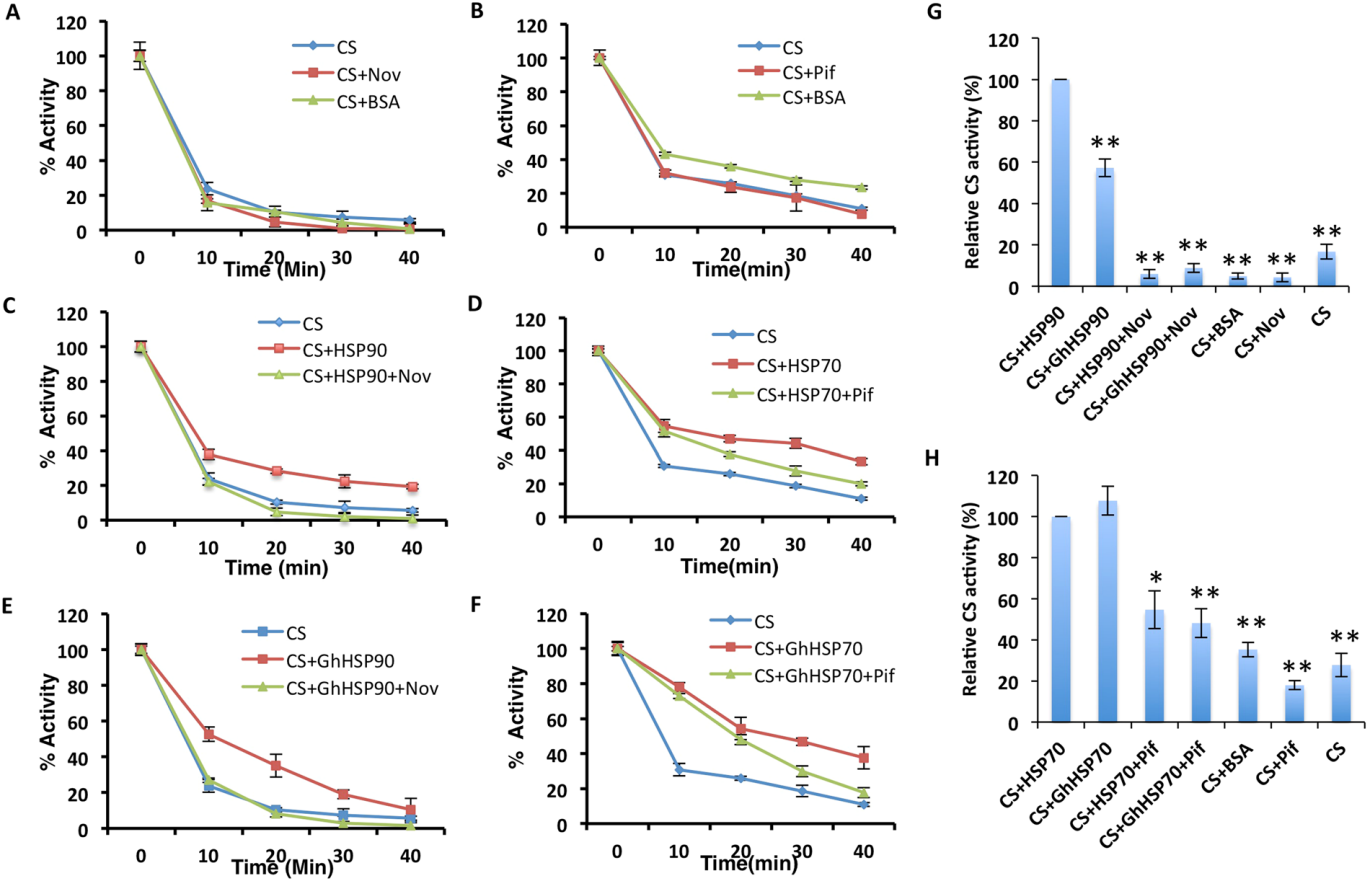
Chaperone assay of GhHSP proteins. (**A**) Effect of control protein BSA and inhibitor Nov on activity of Citrate synthase (CS) (**B**) Effect of control protein BSA and Pif on activity of CS. (**C**) Effect of Human HSP90 on activity of CS, with or without Nov. (**D**) Effect of Human HSP70 on activity of CS, with or without Pif. (**E**) Effect of GhHSP90 on activity of CS, with or without Nov. (**F**) Effect of GhHSP70 on activity of CS, with or without Pif. (**G**) The mean CS activity at the end point of A, C and E relative to CS-thermosprotection in the presence of HSP90 protein. (**H**) The mean CS activity at the end point of B, D and F relative to CS-thermosprotection in the presence of HSP70 protein. The asterisks represent statistical significance between two independent experiments (*p-value ≤ 0.05, **p-value ≤ 0.01).

The specificity of inhibition of fiber development by Nov and Pif was evaluated using Citrate synthase (CS) assay^35^. CS is a substrate for both HSP90 and HSP70, and it is a thermally unstable protein that makes it suitable for analyzing chaperone activities^35,36^. Initially, the assay was standardized using human CS, HSP90 and HSP70 obtained from Sigma, USA (Fig. 3A–D). The Nov/Pif (Fig. 3A and B) alone do not affect the activities of CS. However, the inclusion of human HSP90 (Fig. 3C) and HSP70 (Fig. 3D) in the reactions showed significant chaperonic thermos-protection as expected. But, BSA at an equivalent concentration does not show thermos-protection on CS (Fig. 3A and B). Further, the addition of Nov to the reaction containing CS and human HSP90 resulted in the loss of thermos-protection by HSP90 (Fig. 3C). Similarly, Pif also inhibits thermos-protection by HSP70 (Fig. 3D). Thus, our results validate previously reported thermos-protection activities of HSP90 and HSP70 on CS^35^ and the specificity of inhibition of these interactions by Nov and Pif respectively. Next, we replaced the human HSPs from the CS thermos-protection assay with bacterially expressed partially purified recombinant GhHSP90-7 and GhHSP70-8 proteins (Supplementary Fig. S4). We observed, similar to human HSP90 and HSP70, GhHSP90-7 and GhHSP70-8 also showed significant thermos-protection to CS in our assay (Fig. 3E and F). Further, the addition of Nov and Pif completely inhibited the thermos-protection activity of GhHSP90-7 and GhHSP70-8, respectively (Fig. 3E and F). Thus, our results showed that inhibition of fiber development observed in *in-vitro* condition by Nov and Pif was indeed due to their inhibitory effect on chaperonic activities of GhHSP90 and GhHSP70, respectively.

### Inhibition of HSP90 and HSP70 activity leads to ROS imbalance and autophagy in developing fibers

**Figure 4:**
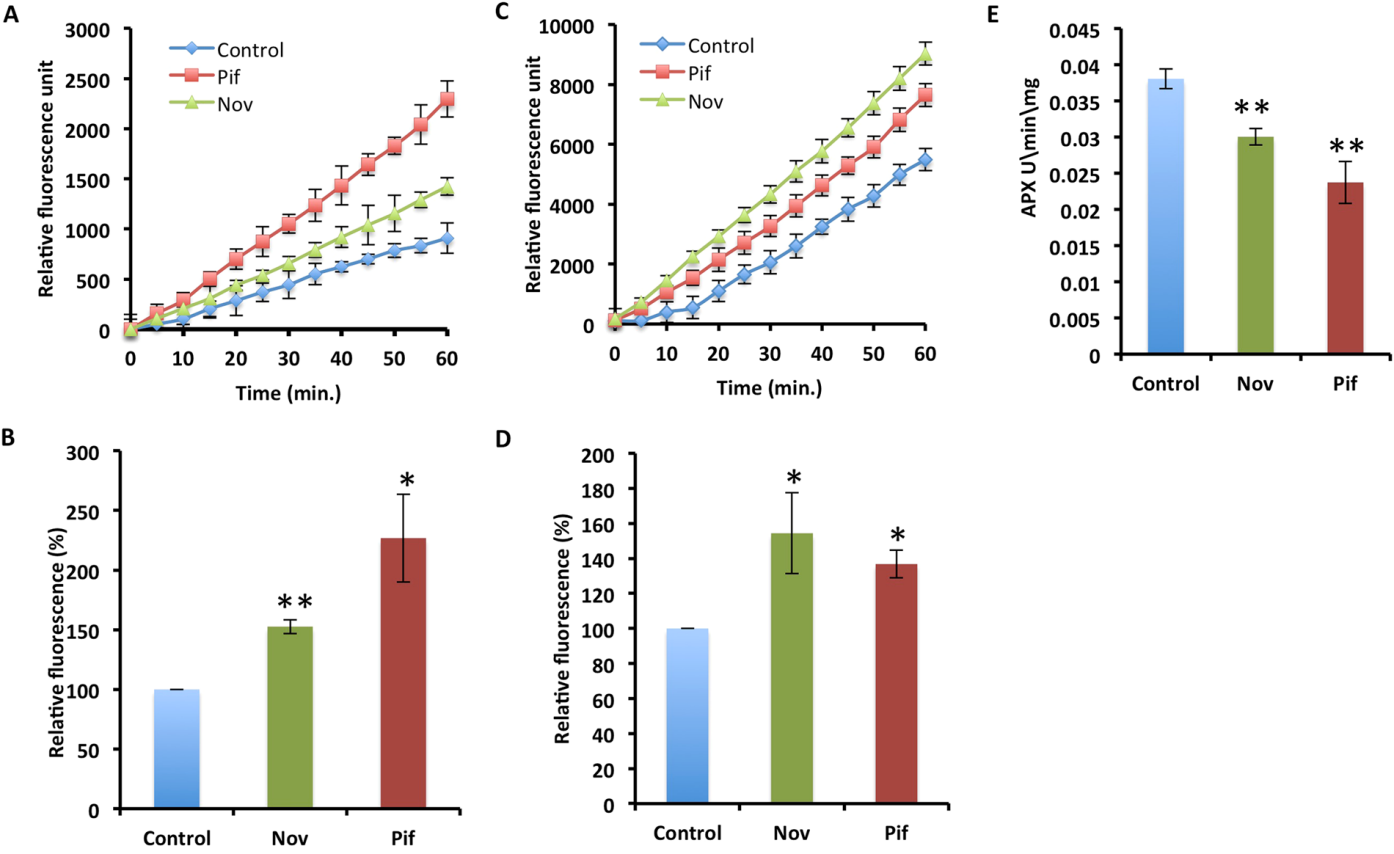
Biochemical alterations due to HSP inhibitor treatment in *in-vitro* cultured ovules. (**A**) Relative H_2_O_2_ estimation in Nov and Pif treated ovules at 0 DPA. (**B**) The mean fluorescence at the end point of Nov and Pif treated ovules as compared to control ovules at 0 DPA. The asterisks represent statistical significance between three biological and two technical replicates (*p-value ≤ 0.05, **p-value ≤ 0.01). (**C**) Relative H_2_O_2_ estimation in Nov and Pif treated ovules at 6 DPA (D) The mean fluorescence at the end point of Nov and Pif treated ovules as compared to control ovules at 6 DPA. The asterisks represent statistical significance between three biological and two technical replicates (*p-value ≤ 0.05, **p-value ≤ 0.01). (**E**) Quantitative estimation of Ascorbate peroxidase (APX) activity in Nov and Pif treated ovules at 0 DPA (**p-value ≤ 0.01).

The HSPs are involved in cellular homeostasis during stress and rapid growth. Thus, any imbalance in HSPs like inhibition of HSP activity by inhibitors impaired cellular homeostasis and therefore resulted in ROS imbalance. Fiber development also requires finely tune ROS response^5^. Thus any imbalance in ROS may lead to improper fiber development. Hence, we examined ROS response in Nov or Pif treated ovules by estimating the H_2_O_2_, superoxide, and ascorbate peroxidase (APX) activity^7,37,38^. The Nov and Pif treated ovules at 0, and 6 Days post anthesis (DPA) in *in-vitro* condition showed a significant increase in H_2_O_2_ and superoxide levels (Fig. 4A,C, and Supplementary Fig. S5). The results suggested that the inhibition of HSPs leads to significantly higher accumulation of H_2_O_2_ and superoxide radicals. Further, as expected treatment of Nov or Pif also leads to significant decrease in the APX activity at 0 DPA, which correlates, well with higher H_2_O_2_ level (Fig. 4E). Thus, results indicate that treatment of developing fibers with Nov and Pif leads to an imbalance in ROS.

**Figure 5:**
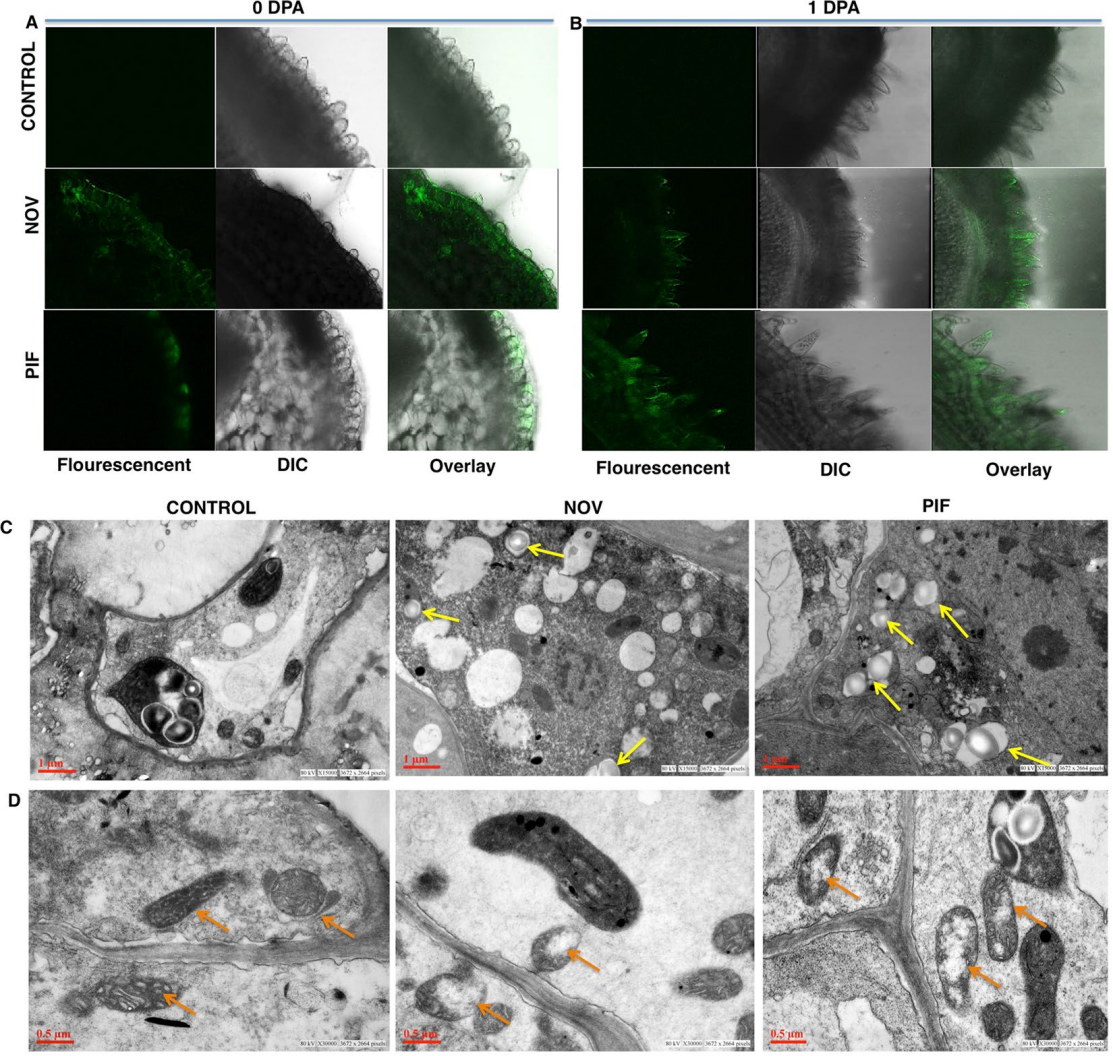
Induction of autophagy in Nov and Pif treated cotton ovules. Monodansylcadaverin (MDC) staining shows the presence of autophagosomes in cotton ovules treated with inhibitors Nov and Pif at (A) 0 DPA (**B**) 1 DPA (**C**) TEM of Nov and Pif treated ovules show the presence of autophagosomes (yellow arrow) at 1 DPA (D) TEM of Nov and Pif treated ovules show the presence of disrupted mitochondrial cristae (orange arrow) in treated ovule at 1 DPA.

The higher accumulation of H_2_O_2_ and inhibition of HSP has shown to destine the cells to autophagy-mediated cell death^39,40^. Thus, we examined the potential induction of autophagy in Nov and Pif treated fibers at 0 DPA and 1 DPA by staining them with monodansylcadaverine (MDC). The committed fiber cells (0 DPA) and the protruding fibers (1 DPA) showed significant fluorescence in the Nov and Pif treated ovules indicating a higher level of autophagosomes, whereas no significant fluorescence was observed in control ovules treated with DMSO (Fig. 5A and B). Thus, our results revealed that higher accumulation of H_2_O_2_ due to inhibition of HSP activities lead to significant induction of autophagy in the fibers treated with Nov and Pif.

The TEM analysis of 1 DPA fibers of control or Nov/Pif treated fiber cells further confirmed the results (Fig. 5C). The TEM revealed that Nov and Pif treated fiber cells showed a significantly higher number of refractive autophagosomes, which are altogether absent from the un-treated fiber cells. The previous report also suggested that autophagy caused due to oxidative stress, targets ROS production sites, such as mitochondria^41^. In TEM images we observed that Nov and Pif treated cells showed abnormal mitochondrion with disorganized cristae while that in control was well formed (Fig. 5D). Thus, results confirm that HSP inhibition causes induction of oxidative stress in the ovule that ultimately leads to autophagy.

### HSP90 and HSP70 inhibition result in modulation of the transcriptome during cotton fiber development

**Figure 6:**
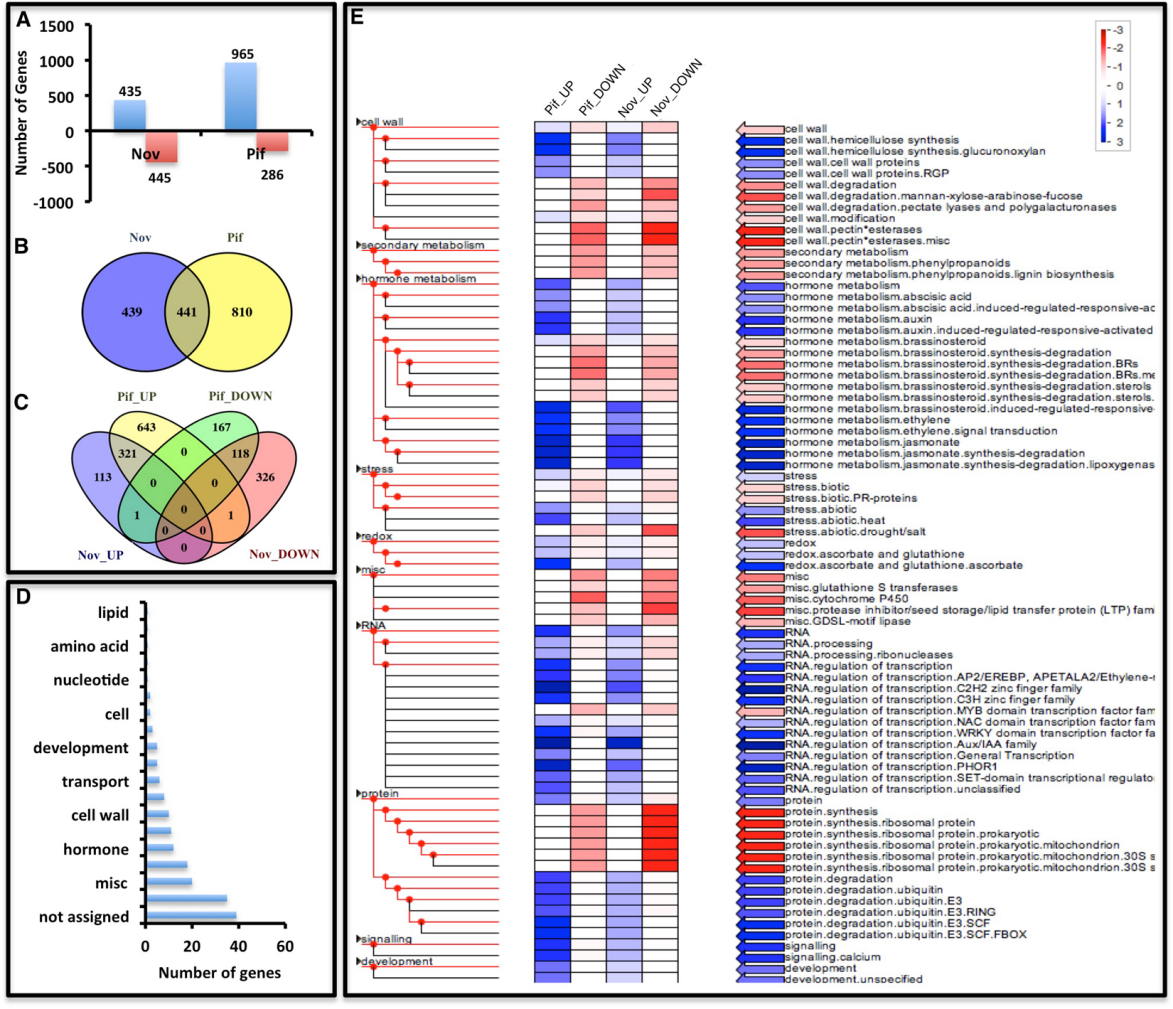
Transcriptomic alterations in HSP90 and HSP70 inhibitor treated *in-vitro* cultured cotton ovules. (**A**) Differentially regulated genes in Nov and Pif treated ovules at 6 DPA. (**B**) Venny diagram showing common genes getting differentially expressed in both Nov and Pif treated ovules. (**C**) Venny diagram showing regulation status of all the DEGs in both the treatments. (**D**) Bar chart showing distribution of common DEGs in different biological processes. (**E**) Pageman showing major pathways regulated by Nov and Pif inhibition in elongation stage.

Cotton fiber development is a complex process as it involves a suite of transcription factors and regulators^1,2^. Application of HSP inhibitors hinders the fiber growth in the developing ovules, which might have accompanied by the pronounced alteration in transcription in developing fibers. The transcriptome sequencing of Nov/Pif treated and control ovules at 6 DPA was carried out (Supplementary Table S2). The quality filtered reads were mapped on *G. hirsutum* reference genome and identified differentially expressed genes (DEGs). Nov treatment leads to up-regulation of 435 genes and down-regulation of 445 genes (total 880 DEGs) as compared to control (Fig. 6A), whereas, Pif treatment results in a total of 1251 DEGs out of which 965 were up-regulated and 286 were down-regulated in 6 DPA ovules (Fig. 6A). The total of 441 DEGs was found to be common between DEGs of Nov and Pif (Fig. 6B) of which 321 DEGs were up-regulated and 118 DEGs were down-regulated in both the cases (Fig. 6C). The percentage of A or D specific expressed genes in either of the inhibitors treated samples remain almost same (Supplementary Table S3). We analyzed common DEGs to identify pathways influenced by inhibition of *HSP90* and *HSP70* during fiber development. It was interesting to note that the DEGs belong to up- and down-class for both the inhibitors were strikingly similar (Fig. 6C). The significant characteristic metabolic bins assigned to DEGs in MapMan analysis are RNA, Protein, hormonal metabolism, signaling, cell wall and stress (Fig. 6D). The pathways and genes that were up-regulated due to inhibition of both HSPs belongs to ABA metabolism, auxin metabolism, ethylene metabolism, jasmonate metabolism, abiotic stress, calcium signaling, GABA amino acid metabolism, nucleotide salvage pathways, vacuole protein targeting, kinases, protein degradation, signaling, miscellaneous pathways like UDP glucosyl and glucoronyl transferases, oxidases, invertase, *AP2/EREBP*, *NAC*, *WRKY*, *AUX/IAA*, *PHOR1*, potassium transporter, armadillo/beta-catenin repeat protein etc. (Fig. 6E, Supplementary Fig. S6). The pathways and genes that were down-regulated include mitochondrial electron transport, cell wall, lignin biosynthesis, brassinosteroid biosynthesis, lipid metabolism, *cytochrome P450*, *GDSL-motif lipase*, *MYB40*, *GST*, *LTP*, protein synthesis, intrinsic protein transporters, etc. (Fig. 6E, Supplementary Fig. S6). These DEGs includes several genes and pathway that were reported to be involved in fiber development such as mitochondrial electron transport, cell wall, phenylpropanoid pathways, brassinosteroid biosynthesis, *cytochrome P450*, *GDSL-motif lipase* and *MYB* transcription factors (Fig. 6E)^42–45^. Besides, HSP inhibition resulted in up-regulation of several stress-related pathways; these include ABA metabolism, auxin metabolism, ethylene metabolism, jasmonate metabolism, abiotic stress, Calcium, *AP2/EREBP*, *NAC*, *WRKY*, *AUX/IAA*, etc. (Fig. 6E)^46–49^. The transcriptome data was further validated using qRT-PCR of seven each of commonly up-regulated (*WRKY53*, *ABA-responsive* gene, *BRH1*, *NAM*, *C2H2-Zn finger*, *Glycoside hydrolase*, and *NDR1*) and down-regulated genes (Ribosomal protein, *WRKY29*, *GDSL-Lipase*, *EXP8*, *UGT72E1*, *MYB40*, and *SQE*) that were identified using RNAseq (Supplementary Table S4). *GbUbiQ1* and *Histone3* were used as internal control genes to normalize the real-time expression values (Supplementary Fig. S7). Several genes that were selected for qRT-PCR validation are implicated earlier for their role in the fiber development. The qRT-PCR analysis showed that all the selected genes showed transcript level similar to that observed in RNAseq, thus validating their expression pattern (Supplementary Fig. S7).

## Discussion

Plants are sessile; therefore, the importance of HSPs in combating stress conditions escalates. HSPs have also been reported to play a crucial role in plant growth and development. Suppression of HSP90 activity leads to developmental defects in *Arabidopsis*^50^, suggesting their essential role in plant development. Likewise, HSP70 is known to bind nascent polypeptides helping them to fold, prevent aggregation and keep them in an import-competent state^51^, also HSP70 has been reported to induce during microspore differentiation in *Capsicum*^52^. Several HSP genes were down-regulated at both RNA and protein levels in *ligon lintless-1* (*Li1*) mutant of *G. hirsutum*, suggesting their involvement in fiber development^29,53^. Cotton fiber development is a complex process, and differential expression of several members of HSPs in the microarray of different fiber developmental stages indicates their involvement in the process (Supplementary Fig. S1). The consistent higher transcript level of HSP90 and HSP70 genes throughout various stages in all the five superior and inferior fiber quality genotypes of *G. hirsutum* suggests their pivotal importance in the development of cotton fiber (Supplementary Fig. S1). Further, the real-time expression analysis of these genes in one of the superior fiber quality genotype of *G. hirsutum* corroborate their role especially in early stages of fiber development, i.e., in fiber initiation and elongation (Supplementary Fig. S2).

HSPs are multigene protein families; different members of HSP gene families might play various roles depending on their pattern of transcript level, substrate specificity, and localization. HSP family members showed differential transcript levels during fiber development (Supplementary Fig. S1). We focused only on *HSP90* and *HSP70* family since they are predominantly expressed during all the stages of fiber development (Supplementary Fig. S1) in *G. hirsutum*. Both the *HSP90* and *HSP70*, gene families, remain highly conserved throughout the kingdom, probably due to their involvement in several fundamental biological processes and defense response. The genome-wide analysis shows the occurrence of 26 and 55 members of *GhHSP90* and *GhHSP70* gene families in *G. hirsutum*, respectively (Tables 1 and 2). The number of these family members are significantly higher in comparison to the other plant species, probably due to tetraploid nature of *G. hirsutum*, but paleopolyploid soybean has yet higher, 61 *GmHSP70* members^54^. The duplication of *GhHSP90-5* and *GhHSP90-7* seems to be evolutionarily relevant, as these genes were also duplicated in the *G. arboreum* (A) and *G. raimondii* (D) progenitors and have transferred to *G. hirsutum* during its speciation. The phylogenetic analysis shows the distribution of *GhHSP90* members into two major groups by their cellular localization (Fig. 1A). The members probably have conserved sub-cellular localization due to their specific functions^55^. *GhHSP70* members present into two major groups in the phylogenetic tree, the higher molecular weight members, *HSP110/SSE* members form one group, and the lower molecular weight members form the other group, probably due to their sub-cellular localization and functional diversity (Fig. 1C). Further, the transcript abundance analysis of *GhHSP90* and *GhHSP70* genes in different stages of fiber development shows the constitutive as well as stage-specific transcript level suggesting the specific and non-overlapping importance of these members. Most of the *HSP90* and *HSP70* homeologs show similar transcript levels and clustering (Fig. 1B and D).

HSPs are chaperone proteins that help in maintaining the protein homeostasis^56^. Cotton fiber development requires a repertoire of genes and proteins expressing during different development stages, the maintenance of cellular homeostasis during the process is a must and may require HSP proteins. Application of HSP90 inhibitor (Radicicol) on developing fibers *in-vitro* hinders fiber elongation, but the study was limited to brief phenotypic observations^29^. Several HSP inhibitors have been extensively studied and characterized in the animal system^32,33^. Application of HSP inhibitors in *in-vitro* ovule culture can prove to be an efficient system for exploring their role in fiber development. We studied the inhibitory activity of two of the previously reported inhibitors of HSP90 and HSP70, i.e., Nov and Pif, respectively in the plant system for the first time. The decline in fiber growth on the application of HSP inhibitors further strengthen the importance of HSP90 and HSP70 activity in both initiation as well as in elongation stage of fiber development. The phenotypic and biochemical parameters suggest varying concentration of Nov and Pif showed pronounced inhibition of fiber development (Fig. 2A and C). The SEM also shows the significant decline in fiber growth (Fig. 2B). However, no significant alteration observed during secondary cell wall deposition stage (Fig. 2C) indicating that HSPs are essential during rapid growth at fiber initiation and elongation.

Mainly the HSP inhibitors for animal HSPs are biochemically characterized till date. However, these HSP inhibitors can potentially inhibit plant derived HSPs, due to their evolutionarily conserved overall domain structure^32,33^. The well-established protocol on thermo-stability of CS in presence or absence of Nov and Pif was used to evaluate their specificity in inhibiting chaperonic activities of GhHSP70 and GhHSP90. Plant-derived GhHSP70 and GhHSP90 showed comparable thermos-protection to the human CS confirming their evolutionary conservation of substrate preference (Fig. 3). The thermos-protecting chaperonic activity of GhHSP70 and GhHSP90 inhibited efficiently by Nov and Pif (Fig. 3E and F), pitching on the importance of these molecules for functional studies on HSPs. Further, the mere addition of Nov or Pif to the reactions do not showed any effect on the activity of CS, indicating that the inhibition by Nov and Pif are specific (Fig. 3A and B). The results thus confirmed that inhibition of fiber development by treatment of Nov/Pif was actually due to the inhibition of chaperonic activities of HSP70/90.

The increased levels of H_2_O_2_ lead to oxidative stress^8^. Also, H_2_O_2_ balance is crucial in growth and development, including cotton fiber development^5^. In agreement with the previous studies, we also observed a rise in the H_2_O_2_ levels due to HSP inhibition during cotton fiber development^57^. The imbalance in H_2_O_2_ levels leads to inhibition of fiber development in both initiation and elongation stage (Fig. 4A and C). The optimal concentration of H_2_O_2_ plays a vital role to destine the cotton ovule epidermal cell to differentiate into a fiber^5^. The outburst of H_2_O_2_ accompanies the transition from initiation to elongation^5,6^. In our study inhibition of HSP90/70 by Nov and Pif might have resulted in the alteration in cellular homeostasis, which leads to oxidative burst (higher levels of H_2_O_2_ and superoxide radicals) and a decline in fiber growth. The expression pattern of *HSP90* and *HSP70* genes might correlate with H_2_O_2_ levels in fiber cells. The *lintless-fuzzless* mutants show low transcript levels of *HSP90* and *HSP70* genes^42^ and also undergo fiber initiation when treated with appropriate concentrations of H_2_O_2_^5^.

The higher H_2_O_2_ levels have been shown to be detrimental to growth and development in plants^9^. Antioxidant enzymes, like APX, maintain the balance of H_2_O_2_. As expected, lower APX activity leads to higher H_2_O_2_ levels in Nov and Pif treated ovules (Fig. 4E), clearly indicating that Nov and Pif treatment leads to oxidative stress like condition in developing fibers. The optimal APX activity is needed for proper development of fibers since *lintless-fuzzless* mutants showed reduced APX activity^7^. However, significantly higher levels of oxidative radicals (H_2_O_2_ and superoxide) and declined APX activity may have destined fiber cells to death since a rise in the H_2_O_2_ signals the cell toward apoptotic pathways, eventually leading to cell death^58^. We show the accumulation of significantly higher level of autophagosome in both Nov and Pif treated fiber cells (Fig. 5). The autophagy induced due to oxidative stress is accompanied by the destruction of ROS generating sites, such as mitochondria^41^. The presence of disrupted cristae in mitochondria of Nov and Pif treated ovules (Fig. 5D) further confirms the induction of autophagic pathways. Thus inhibition of HSPs leads to failure in maintenance of homeostasis that pushes developing fiber cells to death by autophagy.

The inhibition of HSP proteins does cause a drastic change in the transcript profile of cotton fiber (Fig. 6 and Supplementary Fig. S6). Interestingly, both the inhibitors seem to target same pathways and genes, as seen by shared DEGs in both the inhibitors (Fig. 6). Thus, results indicate that HSP70 and HSP90 may have many common targets, which could be the master regulators of transcription during fiber development. Inhibition of HSPs has been reported to induce oxidative stress in the living system^57^. Our transcriptome data suggest that inhibitor-treated cotton ovules result in differential regulation of several known stress-related genes. *AP2/EREBP* transcription factors have been reported to regulate developmental, physiological and biochemical responses during different stress conditions in plants^48^. *AP2/EREBP* transcription factors, like *CRF2* (cold inducible), *DREB1D* (dehydration and cold-inducible)^48^ were identified as up-regulated in our analysis whereas *RAP2.3* was down-regulated^59^. The C3H type transcription factor such as, *CZF1* which is a salt inducible transcription factor^60^ was identified as up-regulated in our transcriptome data. Several *NAC* family transcription factors that are reported in stress response^61^ were also up-regulated in our data. Ethylene signal transduction is crucial for fiber development as well as in stress response, ethylene signaling related genes such as *ERF5* and *ERF9*^62,63^ were seen up-regulated in the present study. Similarly, *WRKY53* was identified as up-regulated in the transcriptome (Supplementary Fig. S7) which has been reported to be overexpressing during drought stress in *Arabidopsis*^49^. Members of C2H2 zinc finger family are also identified as up-regulated, such as *ZAT10* and *ZAT12* (cold inducible). We observed up-regulation of the genes belonging to ABA metabolic pathways known to have a critical role in stress hormone in plants^64^. Calcium acts as an essential signaling molecule in both fiber development and in coping with stress. Enhanced H_2_O_2_ levels also induce Ca^2+^ signaling pathways^65^, the rise in Ca^2+^ signaling pathways in the present study might be due to the high concentration of H_2_O_2_ in inhibitor-treated ovules (Fig. 6). All the genes and pathways that were up-regulated points towards the activation of multiple stress-related pathways during HSP inhibition. Inhibition of HSPs facilitates protein degradation via ubiquitin-mediated pathways^66^. In concordance, we identified up-regulation of several genes involved in ubiquitin-mediated protein degradation. Similarly, protein degradation related *PHOR1* transcription factors were also up-regulated^67^. Thus, results indicate that HSP inhibition by treatment with inhibitors results in improper protein folding and that may lead the proteins towards ubiquitin-mediated protein degradation pathways in cotton fiber.

Apart from up-regulated genes, there were several genes that are reported to play a role in fiber elongation, such as *Pectate lyase*^68^, *WRKY*^69^, *GDSL-lipase*^70^, lignin biosynthesis related gene *UGT72E1*^71^ were down-regulated in the present study. Further, *Cytochrome p450* genes which are involved in brassinosteroid biosynthesis and having an important role in fiber elongation were down-regulated in our study^72^. Expansins are the cell wall loosening enzymes which are important for fiber elongation^73^ were found to be down-regulated in the present study. Thus major conclusion from transcriptome indicates that inhibition of HSPs leads to up-regulation of genes and pathways that are involved in managing stress and down-regulation of several genes reported to play an essential role in fiber development.

Thus, our study points towards the importance of *HSP90* and *HSP70* in fiber initiation and elongation. HSP90 and HSP70 inhibition lead to oxidative stress and autophagic cell death in initiating and elongating fiber cells. We observed up-regulation of genes belonging to several stress-related pathways and down-regulation of several fiber-elongation related genes concurrently to inhibition of HSP90 and HSP70. Our study thus points out the importance of chaperone and their possible engineering for better fiber yield and quality.

## Material and Methods

### Plant Materials

*G. hirsutum* genotype JKC725 was used in the present study for all the experimental purposes. Ovules from field grown plants were excised at −3 and 0 DPA for *in-vitro* ovule culture. The 6 DPA ovules were used for RNA extraction and full-length cloning of *GhHSP90* and *GhHSP70*.

### Microarray data retrieval and transcript level analysis of HSPs

Cotton fiber in-house microarray data (GSE36228) from our previous study^34^ was used to perform gene expression analysis at different stages of fiber development (0, 6, 9, 12, 19 and 25 DPA). Based on the annotation of probe sets, log_2_ expression values of all the HSP genes were fetched (p-value ≤ 0.05). A heat map was generated to visualize the transcript level of significantly expressed HSP genes using MeV v2.0 software (http://mev.tm4.org/#/welcome).

### Gene family, phylogenetic tree and transcript level analysis

The *Arabidopsis* HSP90 and HSP70 protein sequences were used as a query to perform BlastP similarity search (ftp://ftp.ncbi.nlm.nih.gov/blast/executables/blast+/LATEST/) against *G. hirsutum* proteome data available at CottonGen database^74^. The sequences obtained were analyzed for the presence of characteristic domains of both the families using Conserved Domain Database (CDD; https://www.ncbi.nlm.nih.gov/Structure/cdd/wrpsb.cgi)^75^. Further, the identified genes were classified as A- and D-subgenome homeologues by using their homology to respective subgenomes. The identified HSP genes were assigned name following the nomenclature pattern in the previous publication^26^. For sub-cellular localization prediction two web-based tools, namely, CELLOv.2.5 and TargetP 1.1 were used. The protein sequence of all the members was aligned using inbuilt ClustalW program of MEGA v6.06 package. HSP90, and HSP70 specific phylogenetic analysis was performed using MEGA v6.06 and the neighbor-joining method with 1000 bootstrap value^76^. To examine the transcript levels of the *HSP90* and *HSP70* gene family members in different fiber developmental stages, publically available transcriptome datasets^5^ were downloaded from NCBI SRA database (Supplementary Table S1) and analyzed using DNASTAR QSeq software^77^. The expression profile of genes was visualized using a heatmap generated using MeV v2.0 software^78^.

### *In-vitro* cotton ovule culture and inhibitor treatment

*In-vitro* ovule culture was performed with cotton ovules excised at −3 DPA for initiation and 0 DPA for elongation related studies using the method described by Beasley and Ting (1973)^79^. In brief, the ovules were surface sterilized with 0.1% HgCl_2_ solution (w/v) and cultured in ½ murashige and skoog liquid (MSL) media supplemented with plant growth hormones, 5 µM α-NAA (Sigma Aldrich) and 0.5 µM gibberellic acid (Sigma Aldrich) and incubated at 32 °C in the dark. HSP90 and HSP70 specific inhibitors, novobiocin (Sigma Aldrich) and pifithrin (Sigma Aldrich) respectively, were used in the present study to elucidate their role in fiber development. The inhibitors were dissolved in dimethyl sulfoxide (DMSO; Sigma Aldrich) and added to cultured ovules at various concentrations (Nov: 6.3, 15.7 and 31.5 µM and Pif: 16, 20 and 24 µM). The effect of HSP inhibition on initiation, elongation and SCW stage, inhibitor treatment was observed on cultured ovules in six biological and three technical replicates at −3, 3 and 14 DPA, respectively. An equal concentration of DMSO was used to treat the control ovules. Cultured ovules were examined for the difference in fiber development under control and inhibited conditions. The inhibitor concentration (IC_50_) value that corresponds to 50% fiber growth inhibition was calculated for both the inhibitors by estimating their TFU. Further, images were taken at 6, 12 and 24 DPA for initiation, elongation and SCW stages, respectively, using Lumix DMC FZ-70 camera (Panasonic).

### Scanning Electron Microscopy

Control and treated *in-vitro* cultured ovules at 1 DPA stage were washed twice with 1XPBS (pH 7.2) followed by thorough washing in 0.1 M sodium cacodylate buffer and fixed in 2.5% glutaraldehyde and 4% paraformaldehyde solution for overnight at 4 °C. Ovules were again washed thrice using 0.1 M sodium cacodylate for 20 min each and then transferred in osmium tetraoxide for overnight. Further, two washings of 0.1 M sodium cacodylate were conducted to remove excess osmium tetraoxide. Next, dehydration was carried out in acetone series using 15%, 30%, 60% and 90% solution. At least 3 changes were made in 100% acetone for 20 min each. Samples were dehydrated till they reach critical point of dehydration (CPD) and finally coated with platinum particles (2 coating). The platinum coated samples were observed under the scanning electron microscope (FEG450 Quanta, Netherland).

### Quantitative estimation of fiber parameters

To observe the effect of inhibitors on fiber growth of *in-vitro* cultured 3 DPA old ovules (initiation stage) and 12 DPA old ovules (elongation stage) was analyzed by estimating TFU^80^. Briefly, treated and control ovules in three biological and three technical replicates were stained in toluidine blue solution, followed by through washing with distilled water. The stained ovules were immersed in the de-staining solution and absorbance of the de-staining solution was monitored (in triplicates) at 624 nm on UV-Vis spectrophotometer (Shimadzu, Japan) after 1 h incubation. The concentration at which the TFU value was half to that of control ovules was designated as IC_50_ value for the corresponding inhibitor. Further, to examine the effect of inhibitors in SCW stage, cellulose content was estimated in control and treated ovules at 24 DPA using Anthrone method^81^.

### Bacterial expression and purification of recombinant GhHSP90-7 and GhHSP70-8 in *E. coli*

Bacterial expression of recombinant GhHSP90-7 and GhHSP70-8 was performed using champion pET-SUMO expression system (Invitrogen). The full-length coding sequence of *GhHSP90-7* (2.1 Kb) and *GhHSP70-8* (1.8 Kb) was amplified with advantage Taq DNA polymerase (Clontech) using gene-specific primers (Supplementary Table S4) and cloned in the pET-SUMO TA-cloning vector. *E. coli* BL21 (RIL) strain was used to express the proteins. Bacterially expressed recombinant proteins were purified using Ni-NTA columns (Qiagen) according to the manufacturer’s protocol and were confirmed on SDS-PAGE followed by immunoblot using anti penta-His antibody (Qiagen).

### Molecular chaperone assay

The chaperone activity of HSP90 and HSP70 proteins was measured by incubating with substrate CS at an elevated temperature, and the first reaction of the citric acid cycle was monitored^35^. In the first reaction of citric acid cycle acetyl-CoA reacts with oxaloacetic acid in the presence of CS to form acetyl-CoA thioester. DTNB oxidizes acetyl-CoA thioester to form a yellow product that is observed spectrophotometrically. The aggregation of 0.5 µM citrate synthase (from Porcine heart; Sigma) was induced at 43 °C in 50 mM HEPES (pH 8.0), with or without 1.8 µM proteins (BSA or HSP90/70 (Human, Sigma) or GhHSP90/70) and with or without HSP90/70 inhibitors Nov or Pif respectively. The activity of CS was monitored spectrophotometrically at 412 nm in the presence of 0.1 mM oxalo acetic acid, 0.1 mM DTNB [5,5’-dithiobis(2-nitrobenzoic acid)] and 0.05 mM acetyl-CoA in TE buffer (50 mM tris, 2 mM EDTA, pH 8.0). The readings recorded at 25 °C at an interval of 10 min for total of 40 min. The experiment was performed twice, each with two biological and three technical replicates.

### Histochemical detection of relative H_2_O_2_ and superoxide in cultured ovules

Relative H_2_O_2_ levels were measured in control and inhibitor-treated ovules at 0 and 6 DPA, using cell-permeable 2′,7′-dichlorodihydrofluorescein diacetate (H_2_DCFDA)^82^. Control and inhibitor-treated cotton ovules in three biological and two technical replicates were incubated in H_2_DCFDA solution (0.5 mg/ml in 1XPBS) at room temperature. Conversion of non-fluorescent H_2_DCFDA to fluorescent 2′,7′-dichlorofluorescein (DCF) in ovules was measured by fluorimeter (BioTek FLX800; excitation/emission: 485/525 nm) for 60 min at the interval of 10 min. For the detection of superoxide radicals, control and treated ovules at 0 and 6 DPA were stained with nitro-blue tetrazolium chloride (NBT) (Roche Diagnostics)^83^. Ovules were stained with NBT staining solution (0.2% NBT in 50 mM sodium phosphate buffer) for 10 min. Followed by through washing with distilled water. The images of ovules were captured under stereo-microscope (Leica MZ 125, Germany).

### Ascorbate peroxidase assay

The control and inhibitor-treated cultured ovules at 0 DPA were crushed in liq. nitrogen and homogenized in 1 ml extraction buffer containing 50 mM phosphate buffer (pH 7.0), 1 mM EDTA, 2% PVP, 10% glycerol and 1 mM ascorbate. The homogenate was centrifuged at 13,000xg for 30 min at 4 °C and supernatant was used for estimation of APX activity by spectrophotometric method^7,84^. Briefly, 100 µl of sample was mixed with assay buffer containing 50 mM phosphate buffer (pH 7.0), 0.1 mM EDTA and 0.5 mM ascorbate. The reaction was initiated by addition of 0.1 mM H_2_O_2_ and change in absorbance was monitored at 290 nm in UV/Vis-spectrophotometer (Perkin-Elmer) for 3 min at 30 seconds interval. The APX activity was calculated by a decrease in absorbance of ascorbate. One unit of enzyme activity was defined as the amount of APX required for the oxidization of 1 μmol ascorbate at 25 °C in 1 min.

### MDC staining

MDC dye was used to stain autophagic vesicles. Ovules at 0 and 1 DPA were stained with a 0.05 mM final concentration of monodansylcadaverine (Sigma) in 1XPBS for 10 min^85^. Ovules were washed twice with 1XPBS to remove excess MDC. Transverse sections of ovule were observed under LSM 510 META confocal microscope (Carl Zeiss), with an excitation wavelength of 335 nm and an emission band pass of 505–535 nm.

### Transmission electron microscopy

The ovules were cultured at −3 DPA and treated with inhibitors. At 1 DPA the control and treated ovules were washed with 1XPBS (pH 7.2). The ovules were then fixed in 2.5% glutaraldehyde prepared in 0.1 M sodium cacodylate buffer (pH 7.2) (Ladd Research) for 4 h at 4 °C followed by three times washing with 0.1 M sodium cacodylate buffer. Further, the ovules were treated with 1% osmium tetraoxide for 4 h and thoroughly washed with sodium cacodylate. After this, the ovules were dehydrated in acetone series (15–100%) and then embedded in araldite-DDSA mixture (Ladd Research Industries, USA) followed by baking at 60 °C. Ultra-microtome (Leica EM UC7) was used to cut 60-80 nm thick sections from the blocks. Further, these sections were stained with uranyl acetate and lead citrate and analyzed under FEI Tecnai G2spirit twin transmission electron microscope equipped with Gatan digital CCD camera (Netherland) at 80 kV.

### RNA extraction from cultured ovules

Total RNA was isolated from control (DMSO), HSP90 and HSP70 inhibitor-treated ovules at 6 DPA using the spectrum plant total RNA isolation kit (Sigma Aldrich). After DNaseI (Ambion) treatment, quality and quantity of samples were checked using 2100 Agilent Bioanalyzer (Agilent) and Nanodrop 1000 spectrophotometer (Thermo Scientific), respectively.

### Transcriptome analysis

Control and inhibitor treated RNA samples at 6 DPA were used for library preparation and sequenced by paired end sequencing method^14^ with Illumina NextSeq. 500 sequencing platform. The fastq files generated after sequencing were quality filtered using FASTX toolkit (http://hannonlab.cshl.edu/fastx_toolkit/) (Supplementary Table S2). The processed reads were aligned with default parameters using Tophat (version 2.1.1) on *G. hirsutum* genome downloaded from Cotton Genome Project (CGP) (http://cgp.genomics.org.cn/page/species/download.jsp?category=hirsutum). The estimation of FPKM (Fragment per kilo per million) values for expression of genes and transcripts were performed using Cufflinks program (version 2.0.0) with default parameters. The DEGs were filtered by p-value ≤0.01, FDR ≤0.05 and log fold change > 1, and these DEGs were used further for pathway analysis using Mapman (version 3.5.1R2).

### qRT-PCR Analysis

First-strand cDNA was synthesized from DNaseI treated total RNA using SuperScript III (Invitrogen). The qRT-PCR reaction was carried out for selected DEGs (Supplementary Table S4) using SYBR Green PCR master mix (Invitrogen) in ABI7500 Fast real-time PCR system (Applied Biosystems). All the reactions were performed in three biological and three technical replicates. *GbUbiQ1* and *Histone3* (Accession number AY375335 and AF024716, respectively)^86^ were used as internal controls for data normalization. Further, average fold change was calculated from the normalized data by using the ∆∆Ct method of ABI7500 SDS software (version 1.2.2).

### Data Availability

Sequence data generated for this study will be available with the NCBI SRA database under the bioproject accession number PRJNA397595.

## Electronic supplementary material

Supplementary Information
