# Supplementary material for: Inhibition of Heat Shock proteins HSP90 and HSP70 induce oxidative stress, suppressing cotton fiber development
Source: Sci Rep. 2018 Feb 26;8:3620. doi: 10.1038/s41598-018-21866-0 (PMC5827756; doi:10.1038/s41598-018-21866-0)
Supplement: Supplementary file 1 — Supplementary Information [file 41598_2018_21866_MOESM1_ESM.pdf]

Inhibition of Heat Shock proteins *HSP90* and *HSP70* induce oxidative stress and suppress cotton fiber development

Anshulika Sable[1,4], Krishan M. Rai[1,2], Amit Choudhary[1], Vikash K. Yadav[1,3], Sudhir K. Agarwal[4], Samir V. Sawant[1]*

The affiliations and addresses of the authors:
[1]Plant Molecular Biology Laboratory, National Botanical Research Institute, Rana Pratap Marg, Lucknow 226001, India
[2]Present address: Department of Plant and Soil Science, Texas Tech University, Lubbock, Texas 79415, USA
[3]Present address: Department of Plant Biology, Uppsala Biocenter, Swedish University of Agricultural Sciences, Uppsala 75007, Sweden
[4]Department of Biochemistry, University of Lucknow, Lucknow 226007, India

Supplementary Information

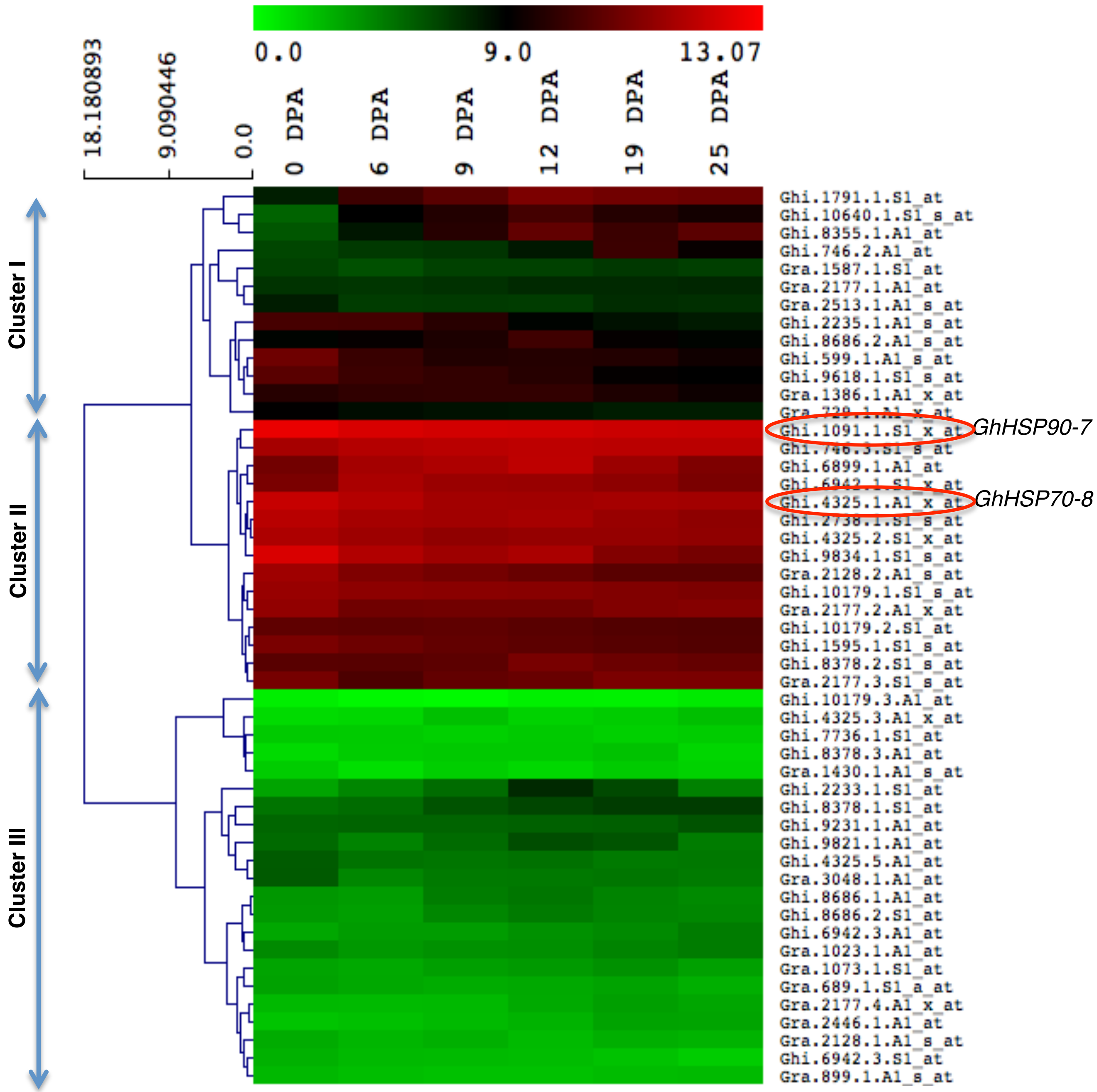

Supplementary Fig. S1. Microarray gene expression profile of HSPs in different stages of fiber development in *G. hirsutum* genotypes. Average transcript level of HSP genes at 0, 6, 9, 12, 19 and 25 DPA in five *G. hirsutum* genotypes. HSPs form medium expressing (Cluster I), high expressing (Cluster II) and low expressing (Cluster III) clusters.

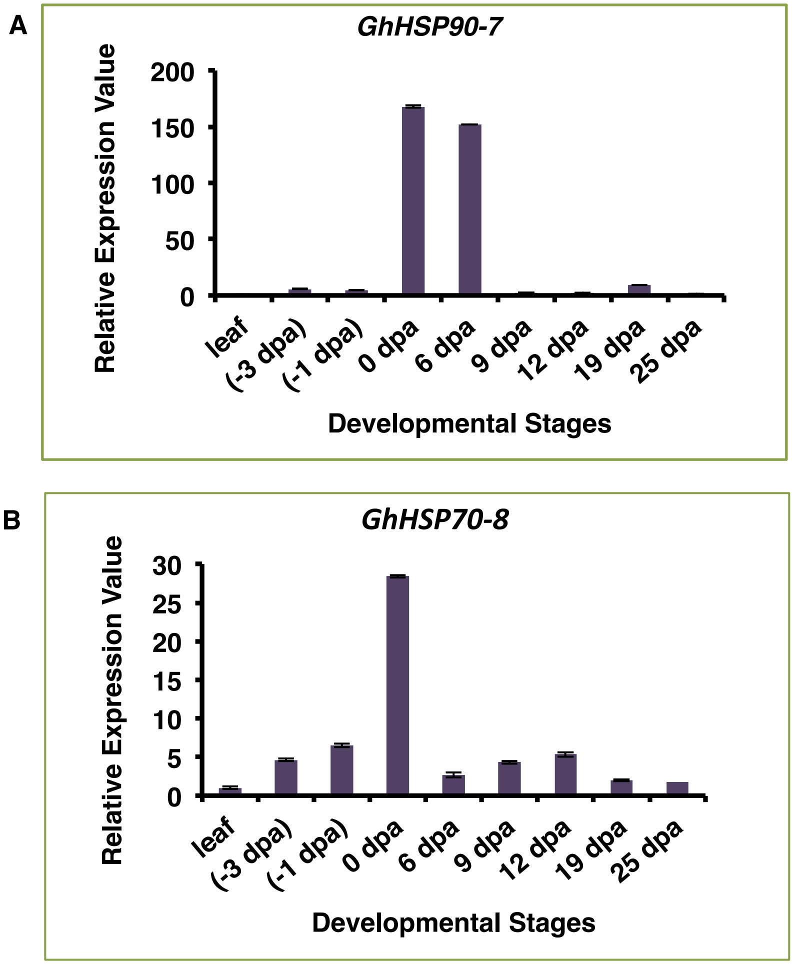

Supplementary Fig. S2. Real time PCR of candidate *GhHSP90-7* and *GhHSP70-8* genes at different fiber developmental stages. (A) *GhHSP90-7* (B) *GhHSP70-8*.

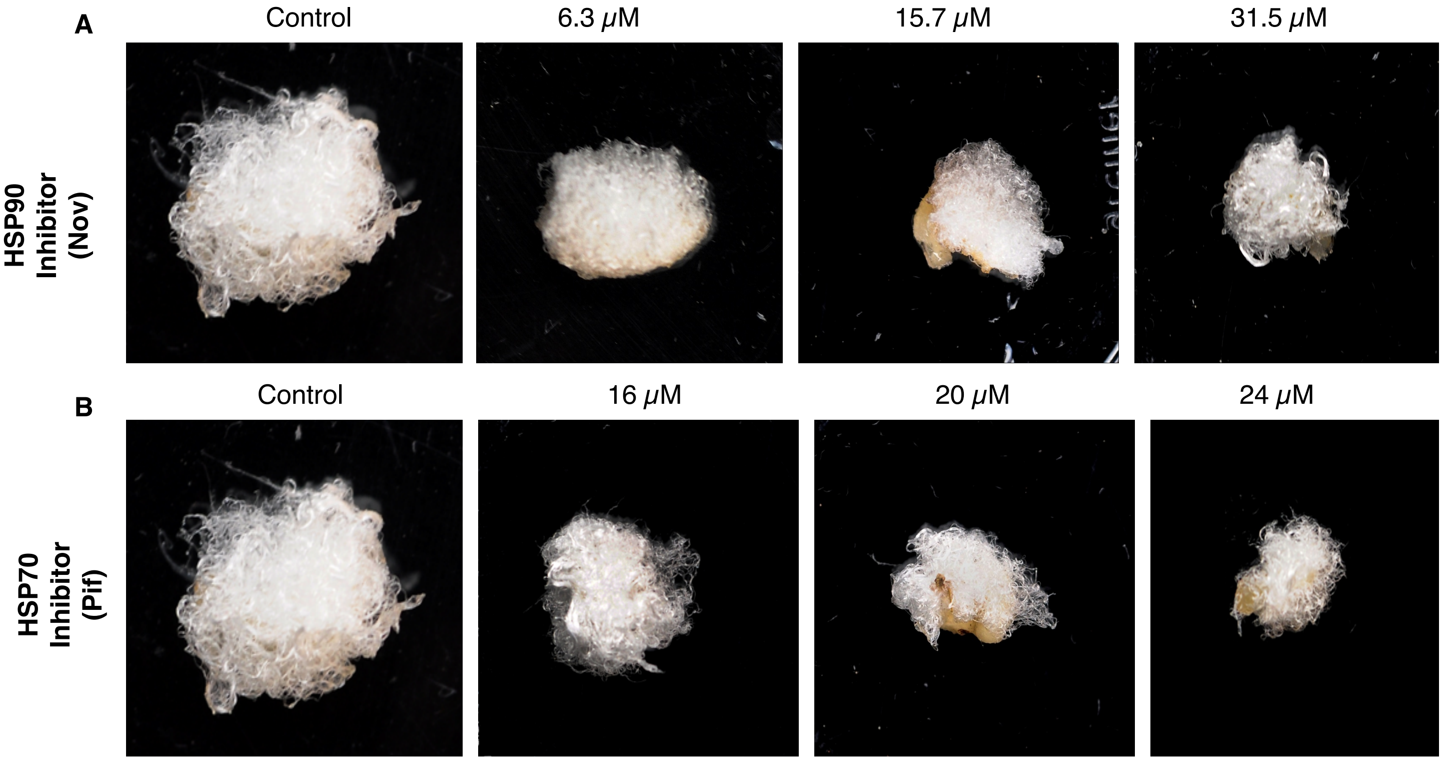

Supplementary Fig. S3. Phenotypic analysis of fiber development at different inhibitor concentrations in *in-vitro* ovule culture. (A) Photographs showing effect of increasing concentrations of Nov (HSP90 inhibitor) in *in-vitro* ovule culture. **(B)** Photographs showing effect of increasing concentrations of Pif (HSP70 inhibitor) in *in-vitro* ovule culture.

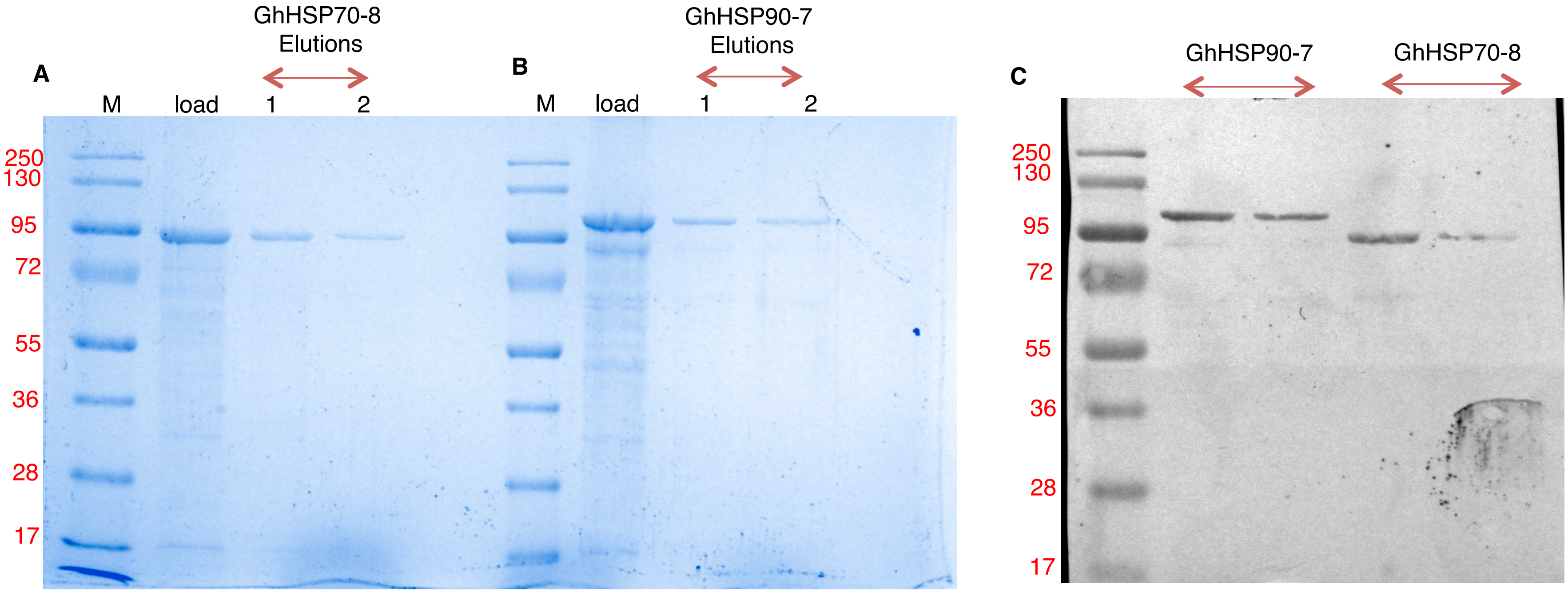

Supplementary Fig. S4. Ni-NTA column purification and immunoblot of bacterial expressed recombinant GhHSP90-7 and GhHSP70-8. (A) SDS-PAGE gel showing bacterially expressed and purified fractions of GhHSP90-7 (B) SDS-PAGE gel showing bacterially expressed and purified fractions of GhHSP70-8. (C) Immunoblot of GhHSP90-7 and GhHSP70-8 purified protein fractions with penta-His antibody.

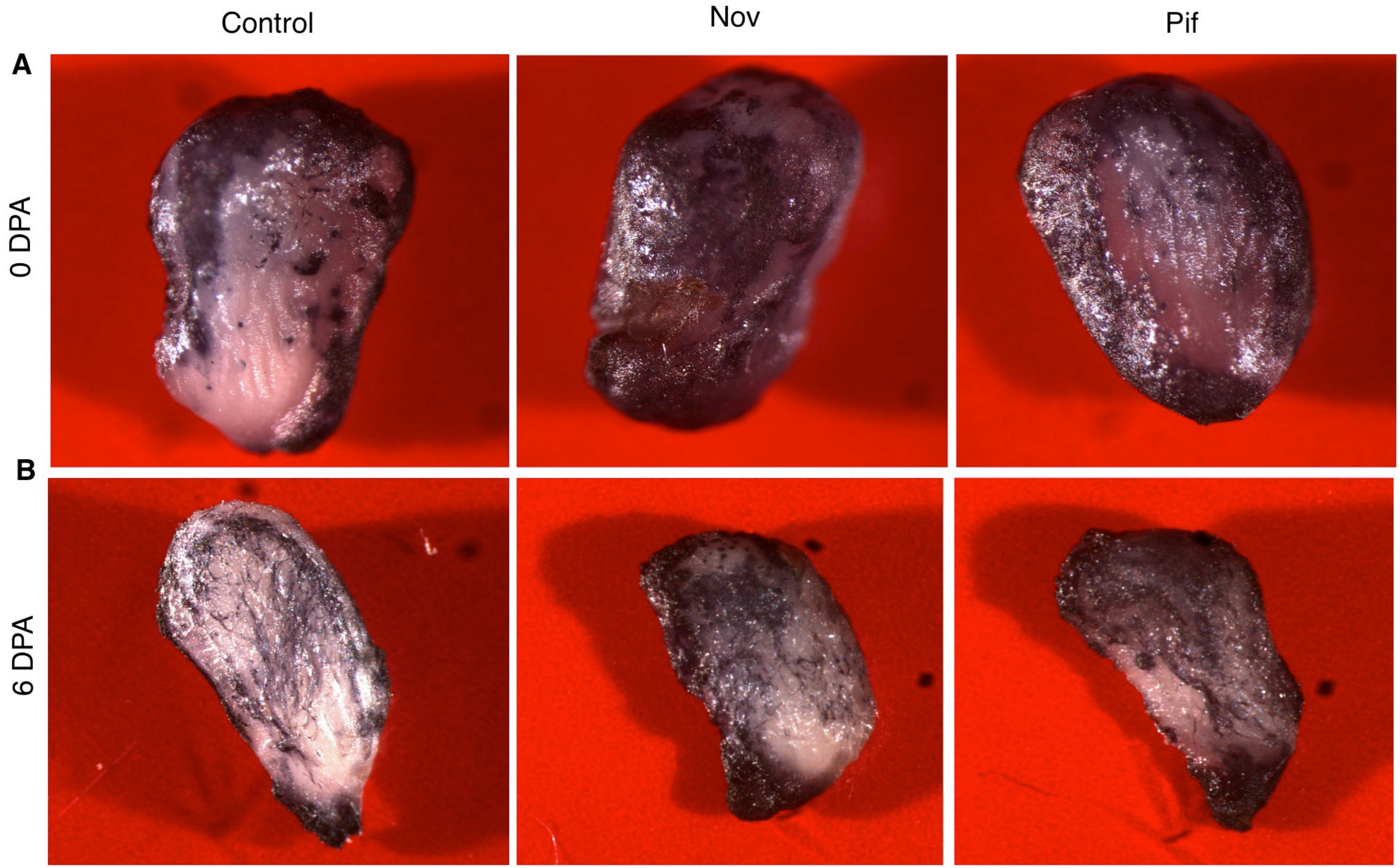

Supplementary Fig. S5. Nitro-blue tetrazolium chloride (NBT) staining showing higher accumulation of superoxide in Nov and Pif treated ovules as compared to control (A) 0 DPA (B) 6 DPA.

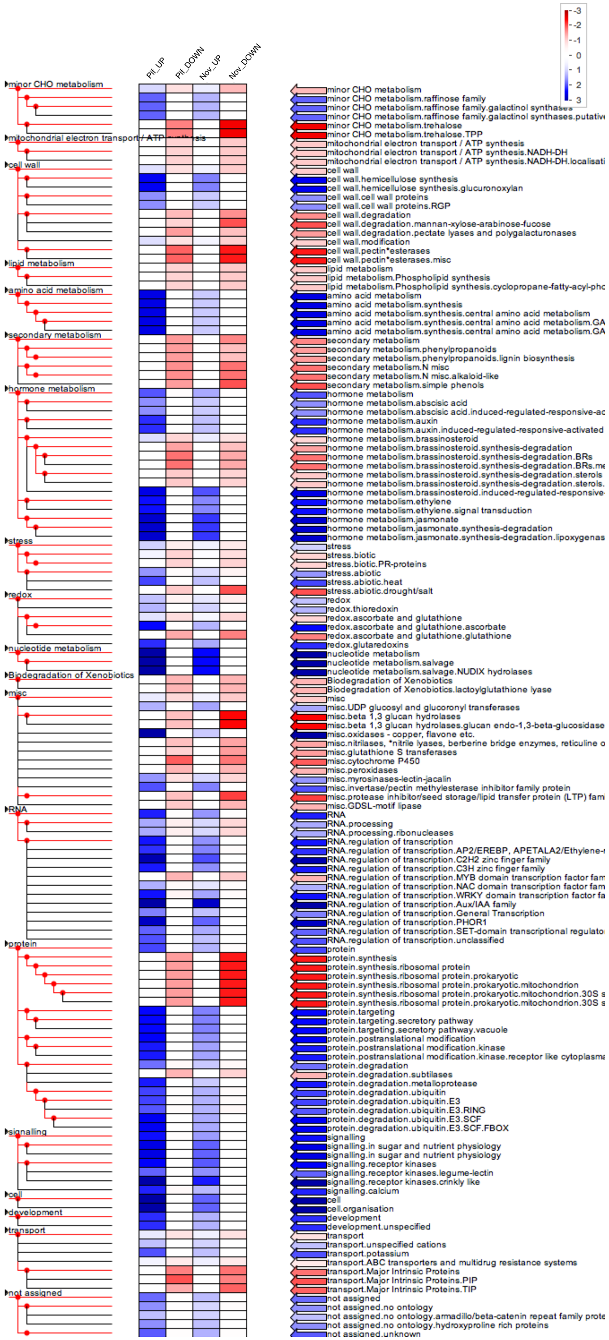

Supplementary Fig. S6. Pageman analysis of common DEGs in HSP90 (Nov) and HSP70 (Pif) inhibited ovules.

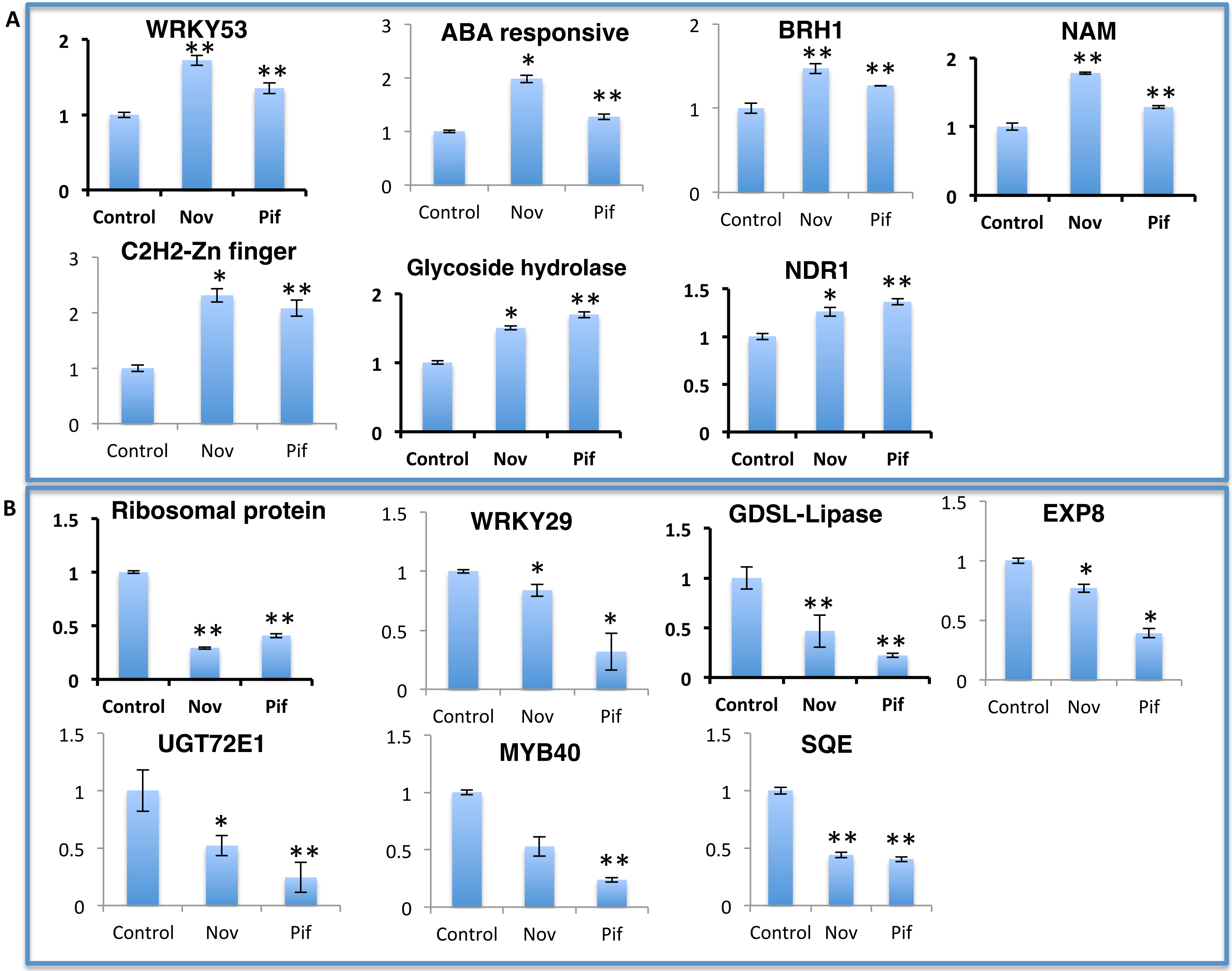

Supplementary Fig. S7. Quantitative real time PCR of randomly selected common DEGs from Nov and Pif treated ovules in three biological and three technical replicates (A) Up-regulated DEGs (B) Down-regulated DEGs. (y-axis denotes the fold-change; *p-value ≤ 0.05; **p-value ≤ 0.01).

Supplementary Tables

Supplementary Table S1. List of publically available cotton fiber transcriptome datasets used for transcript level analysis.

Dataset	Development stage
SRX7697907	-3 DPA
SRX7697908	-1 DPA
SRX7697909	0 DPA
SRX7697910	1 DPA
SRX7697911	3 DPA
SRX7697917	5 DPA
SRX7697918	10 DPA
SRX7697919	20 DPA
SRX7697920	25 DPA

Supplementary Table S2. Transcriptome analysis of inhibitor treated ovules at elongation stage.

Sample_Id	Total_reads	Reads_Aligned	%_Reads_Alignment
Control-1	39173030	31022206	79.2%
Control-2	28424260	22542512	79.3%
Pif-1	24184514	19284706	79.7%
Pif-2	28499642	22692875	79.6%
Nov-1	30231882	24151456	79.9%
Nov-2	26129174	20786511	79.6%

Supplementary Table S3. Allelic contribution of DEGs in transcriptome.

	**HSP90**		**HSP70**	
	**Up (%)**	**Down (%)**	**Up (%)**	**Down (%)**
**A-subgenome**	33.019	28.959	32.432	29.682
**D-subgenome**	45.283	45.023	44.906	39.929
**Scaffold**	21.698	26.018	22.661	30.389

Supplementary Table S4. Primers used in this study.

S. No.	Primer name	Sequence (5'—3')
**A.**	**For cloning into bacterial expression vector**	
1	*GhHSP90-7_F*	ATGGCGGATACTGAGACGTTTGC
2	*GhHSP90-7_R*	TTAATCAACTTCCTCCATCTTGCTG
3	*GhHSP70-8_F*	ATGGCCGGAAAAGGAGAAGGAC
4	*GhHSP70-8_R*	TTAGTCGACTTCTTCTATCTTAGGTCCAGC
**B.**	**For Real time PCR**	
5	*WRKY53_F*	GCAGCCCTCGGAGTGATGA
6	*WRKY53_R*	ACTCTCACATGATCCGTCCATCTC
7	*ABA responsive_F*	ATTGTGACAGTGGATGGACATGATT
8	*ABA responsive_R*	TTGCTACAGAACTGGAAACACTCTCA
9	*BRH1_F*	CCGCTGGATGGATCACGAT
10	*BRH1_R*	TAAACTCAGCTTGCATCTCATCAGGTA
11	*NAM_F*	GGGAGGAAAACCAAATGGAAGA
12	*NAM_R*	TCAACTTAGGAGGAGCAGACACATATG
13	*C2H2 Zn-finger_F*	TCCATTTGCGGCCAAGAGTT
14	*C2H2 Zn-finger_R*	ACAAGCGGAAACGGTGAGAAG
15	*Glycoside hydrolase_F*	CCGTGGATGATACACCCATTAGAG
16	*Glycoside hydrolase_R*	TAAATTCTCATGGGCTGGTTCTTG
17	*NDR1_F*	CCAAACGATACTACCTTAAACCTGACCTT
18	*NDR1_R*	GCGACTTTCACAGCATCATACTTAATC
19	*Ribosomal protein_F*	CCGAAGAAATGGTAAGATATGGGTAAG
20	*Ribosomal protein_R*	GATTTCCTTTCCCTCTTCCCATTC
21	*WRKY29_F*	CATGTTGATGAACCAGCAGAGAAA
22	*WRKY29_R*	TGCTCTACCACTCTATTCTGCTGATTC
23	*GDSL-lipase_F*	GGATGTGCGCCGAAGGA
24	*GDSL-lipase_R*	GTTGTGGACGCGGAAGCAT
25	*EXP8_F*	ATGGTGGCTGGTGCAATCCT
26	*EXP8_R*	AGCCCGGTACTGAGCAATTTG
27	*UGT72E1_F*	GGTGCTCGAACTCGGAAAAAGA
28	*UGT72E1_R*	CAGGAGTTGGGATCGAGACAAA
29	*MYB40_F*	ACCGTGGACGATCGAGGAA
30	*MYB40_R*	CCACACCTTAACAACCCTGCAA
31	*SQE1_F*	ATGGACGTCGAGTGCAAGTGA
32	*SQE1_R*	GCCCCCTGGTTGTAGCAATTC
33	*GbUbiQ1_F*	AGCTCGGATACGATTGATAACG
34	*GbUbiQ1_R*	GAAGACGAAGAACAAGGGGAAG
35	*Histone3_F*	TCAAGACTGATTTGCGTTTCCA
36	*Histone3_R*	GCGCAAAGGTTGGTGTCTTC
